# Progress of Bromophenols in Marine Algae from 2011 to 2020: Structure, Bioactivities, and Applications

**DOI:** 10.3390/md18080411

**Published:** 2020-08-04

**Authors:** Hui Dong, Songtao Dong, Poul Erik Hansen, Dimitrios Stagos, Xiukun Lin, Ming Liu

**Affiliations:** 1 Key Laboratory of Marine Drugs, Ministry of Education of China, School of Medicine and Pharmacy, Ocean University of China, 5 Yushan Road, Qingdao 266003, China; 21190811109@stu.ouc.edu.cn (H.D.); 21190831116@stu.ouc.edu.cn (S.D.); 2 Laboratory for Marine Drugs and Bioproducts of Qingdao National Laboratory for Marine Science and Technology, Qingdao 266237, China; 3 Department of Science and Environment, Roskilde University, 4000 Roskilde, Denmark; poulerik@ruc.dk; 4 Department of Biochemistry and Biotechnology, School of Health Sciences, University of Thessaly, Biopolis, 41500 Larissa, Greece; stagkos@med.uth.gr; 5 Department of Pharmacology, School of Pharmacy, Southwest Medical University, 319 Zhongshan Road, Jiangyang, Luzhou 646000, China; xiukunlin@126.com

**Keywords:** bromophenols, derivatives, marine algae, bioactivity

## Abstract

Marine algae contain various bromophenols that have been shown to possess a variety of biological activities, including antiradical, antimicrobial, anticancer, antidiabetic, anti-inflammatory effects, and so on. Here, we briefly review the recent progress of these marine algae biomaterials and their derivatives from 2011 to 2020, with respect to structure, bioactivities, and their potential application as pharmaceuticals.

## 1. Introduction

Marine algae are exceptionally rich sources of structurally diverse bioactive compounds, and a number of novel compounds exhibiting interesting biological activities have been isolated from marine algae [1,2]. Bromophenols (BPs) are one group of such compounds isolated from marine algae. Structurally, BPs include one or more benzene rings and contain different numbers of hydroxyl, bromine, or other groups. In 1967, the first two BPs were isolated from the red algae *Rhodomela larix* [3]. Over the past few decades, a great number of BPs were isolated from various marine algae species including red algae [4,5,6,7,8,9], green algae [10,11,12,13,14], and brown algae [15,16,17,18,19]. Some BPs are also found in other marine organisms such as sponges [18,20,21,22,23,24], ascidians [25,26,27], mussels [28], polychaetes [29], and marine proteobacteria [30]. However, BPs from microalgae have not yet been reported. It is generally believed that the ecological function of marine BPs is chemical defense and as deterrents for other marine organisms [31].

BPs are common marine secondary metabolites, and biosynthesized in the presence of bromoperoxidases, bromase, laccase, hydrogen peroxide, and bromide. The biosynthetic pathways of these natural BPs are not very clear, with only a few reports illustrating genetic, molecular basis, and bromase effects for the production of BPs in marine organisms [30,32,33,34]. Lindqvist et al. [35] found an apparent correlation between the concentration of pigments in *Ceramium tenuicorne* and the levels of BPs and speculated that this was linked to photosynthetic activity via bromoperoxidase working as a scavenger for the formed hydrogen peroxide. This could be supported by the bromination of 2,4,6-tribromophenol in isolated clonal material of *C. tenuicorne* [36]. Another study of the Chinese marine red alga *Symphyocladia latiuscula* revealed a plausible and convergent biosynthetic pathway of some BPs with a long, branched chain. These kinds of BPs could be regarded as adducts between aconitates and intermediate quinone methide, which were generated from 2,3,6-tribromo-4,5-dihydroxybenzyl alcohol, and aconitic acid was the dehydration product of citric acid. After the intermediate was formed, BPs were produced through reactions such as decarboxylation and oxidation [37].

Over the years, marine BPs have attracted much attention in the field of food and pharmaceutical agents due to their beneficial biological activities [31]. In our previous review, we have summarized the diverse biological activities, such as antimicrobial, anticancer, antioxidant, and antidiabetic, of marine BPs from seaweeds reported before 2011 [38]. In the last decade, novel BPs and more of their bioactivities have been reported. Moreover, it is worth noting that more studies have focused on the design and synthesis of novel functional derivatives of BPs [39,40,41,42,43,44,45,46], as well as on the illustration of the mechanisms underlying their bioactivity, than only focusing on the isolation and testing the biological activity of BPs found in natural products. Therefore, it is necessary to report these new findings, and so in the present review, we mainly focus on the biological activities of newly discovered BPs from marine algae and also on some of BPs’ representative derivatives synthesized in the last ten years. Recently, BPs have also been treated briefly in other reviews [47,48,49].

## 2. Bioactivities of BPs and Potential Use in Medicine

### 2.1. Anticancer Activity

**Scheme 1:**
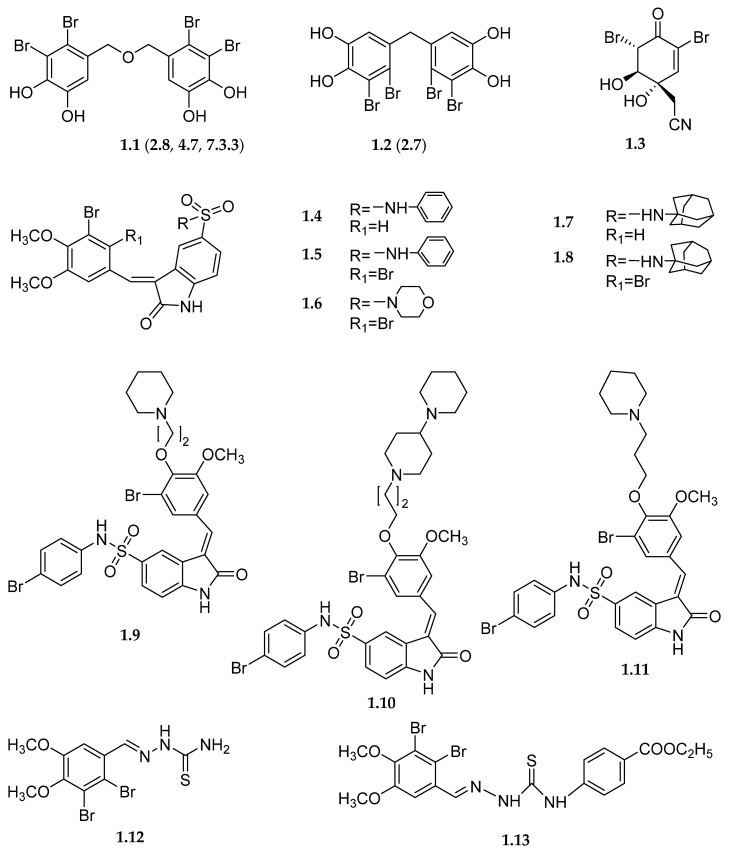
Chemical structures of BPs with anticancer activity.

In the past decade a lot of BPs with anticancer activities have been isolated from marine sources, and some of them possess potential to be developed as novel anticancer agents. For example, BPs isolated from the marine algae *Leathesia nana* and *Rhodomela confervoides* (**1.1** (also **2.8**, **4.7**, and **7.3.3**) and **1.2** (also **2.7** and Scheme 1), respectively) are good candidates as anticancer agents via inhibition of cell growth against various human cancer cell lines and inhibition against the tumor angiogenesis. BP **1.1** was reported to be cytotoxic to human cervical carcinoma cell line HeLa, human colorectal carcinoma cell line HCT-116, human colorectal carcinoma cell line HCT-8, human hepatocellular carcinoma cell line SMMC-7721, human lung adenocarcinoma epithelial cell line A549, and especially to human myelogenous leukemia cell line K562 (Table 1). Compound **1.1** was able to induce mitochondrial apoptosis in cancer cells and inhibit the activity of topoisomerase [50]. However, it seems that BP **1.1** interacts with the DNA molecules rather than directly binding to the topoisomerases [50,51]. Compound **1.1** displayed potent anti-angiogenic activity via disturbing the VEGF signaling [52]. This compound could decrease the migration and cord formation of human umbilical vein endothelial cell line HUVEC and inhibit the subintestinal vessel in zebrafish embryos. Compound **1.2** was able to inhibit the growth of several cancer cells in vitro, including that of HeLa, human colon adenocarcinoma cell line RKO, HCT-116, human glioma cell line U87, and especially of human hepatoma cell line Bel7402 (IC_50_ = 8.7 µg/mL, Table 1). Recent research showed that the in vitro cytotoxic mechanisms of **1.2** were related to the modulation of β1-integrin/FAK signaling and subsequent inhibition of the proliferation, migration, and invasion of hepatocellular carcinoma cells [53]. Moreover, compound **1.2** had anti-angiogenic properties (Table 1) via inhibiting tyrosine kinase and endothelial nitric oxide synthase [8], suggesting anticancer activity as well [53]. Another BP **1.3** (Scheme 1), a dibromotyrosine derivative from the *Pseudoceratina sp.* sponge, also exhibited potent pro-apoptotic effects (Table 1) through targeting the IKK/NFκB signaling pathway as well as inhibiting the activity of topoisomerase II [54].

In order to increase the anticancer potency, synthetic modifications are applied to several marine BPs. Some synthetic derivatives containing an indolin-2-one moiety have been designed, synthesized, and evaluated for their cytotoxic activity. BPs **1.4–1.8** (Scheme 1) containing the indolin-2-one moiety displayed potent cytotoxicity to A549, Bel7402, human hepatocellular carcinoma cell line HepG2, HeLa, and HCT-116 cancer cell lines (IC_50_ values of these BP derivatives against these cancer cell lines are shown in Table 1) [55]. BP **1.9** (Scheme 1) could inhibit the proliferation of many human cancer cell lines, including A549, HepG2, Bel7402, HCT-116 and human clonal colon adenocarcinoma cell line Caco2, with IC_50_ values shown in Table 1. Furthermore, BP **1.9** has been shown to induce cell cycle arrest at the G0/G1 phase of A549 cells and stimulate reactive oxygen species (ROS)-mediated apoptosis in A549 cells [56].

Another BP derivative, **1.10** (Scheme 1) containing the indole-2-one moiety with a similar chemical structure as **1.9**, effectively inhibited the proliferation of human A549 lung cancer cells, with an IC_50_ value of 4.29 ± 0.79 µM. BP **1.10** could block A549 cells at the G0/G1 phase and induce apoptosis [57]. Regarding the molecular mechanisms of this cytotoxic activity, BP **1.10** has been reported to increase the production of cellular ROS and inactivate the PI3K/Akt pathway while activating the mitogen-activated protein kinase (MAPK) pathway [57]. BP **1.11** (Scheme 1), with a similar chemical structure of **1.9** and **1.10**, also showed similar cytotoxic activities against A549 cells through inhibiting the proliferation, arresting G0/G1 cell phase, inducing apoptosis and autophagy via deactivating PI3K/Akt/mTOR, and activating MAPK signaling pathways [58].

Several BP thiosemicarbazone hybrid derivatives with anticancer effects were synthesized in recent years. A novel synthesized BP thiosemicarbazone hybrid **1.12** (Scheme 1) could effectively inhibit human ovarian cancer cell line SK-OV-3, Bel7402, and HepG2 proliferation, with IC_50_ values shown in Table 1, and the compound could induce apoptosis and cell cycle arrest in SK-OV-3 cell line. BP **1.12** also exhibited tumor growth inhibition in an in vivo SK-OV-3 cell xenograft mice model. Further investigation showed that this compound exerted its cytotoxic activity through multiple anticancer mechanisms, such as selective inhibition of poly (ADP-ribose) polymerase-1 (PARP-1) activity, DNA repair alterations, inhibition of PARylation, as well as the production of cytotoxic ROS [59]. In addition, BP **1.12** displayed good pharmacokinetic characteristics and nontoxic behavior [59]. This observation indicated that compound **1.12** may serve as a lead compound for the development of new anticancer drugs. Another BP thiosemicarbazone hybrid **1.13** (Scheme 1) also inhibited PARP-1 (IC_50_ = 58.3 nM) and cell proliferation, induced cells apoptosis, and arrested cell cycle in human breast cancer cell line HCC-1937 [60].

Although the above BPs showed great cytotoxicity, their selectivity is unclear—that is, their effects on normal cells still need to be investigated. In addition, the mechanisms of their anticancer effects have not been fully clarified and their structure-activity (SAR) relationship also needs to be explained further. All this is important in order to find novel and safe compounds as anticancer candidates.

### 2.2. Antidiabetic and Anti-Obesity Activity

**Scheme 2:**
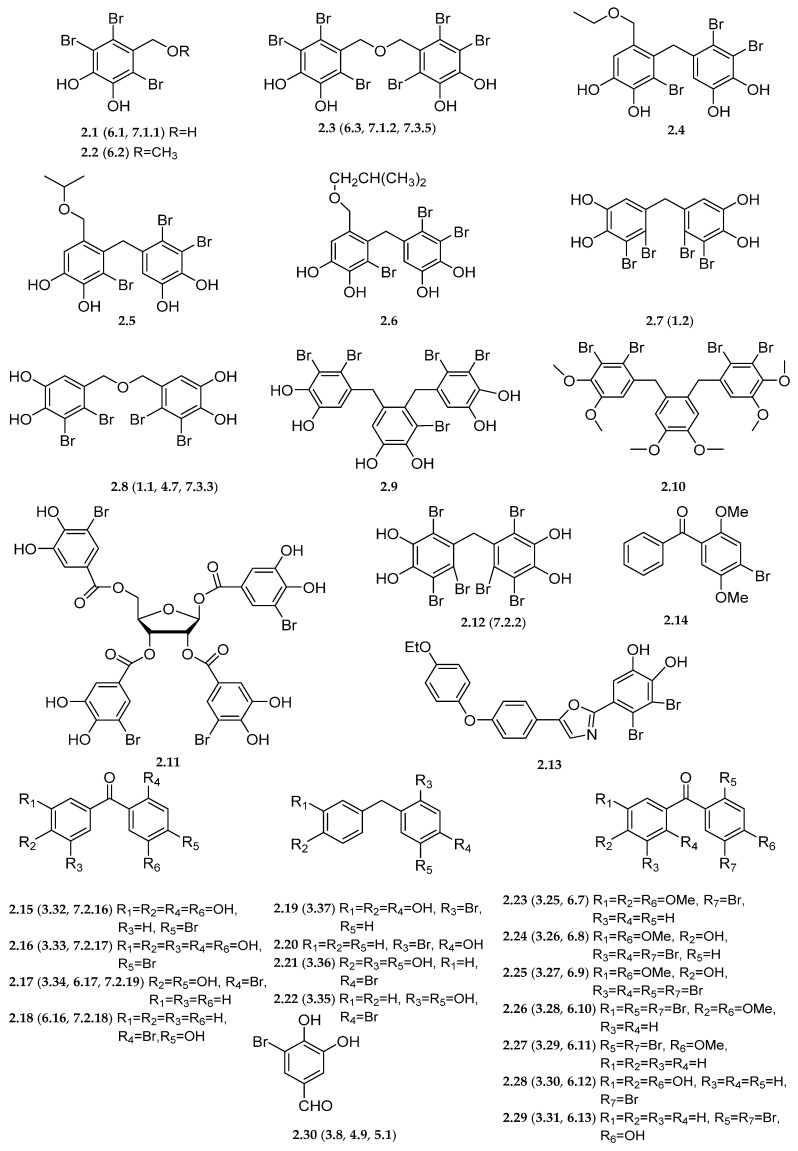
BPs with antidiabetic activity.

Marine algae have been used for a long time as a remedy for diabetes in folk medicine in China [61], and BPs obtained from marine algae have been reported to be potential hypoglycemic agents, acting as inhibitors against metabolic enzymes, mainly including tyrosine phosphatase 1B (PTP1B), α-glucosidase, α-amylase, and aldose reductase (AR). PTP1B is a negative regulator of insulin signaling via dephosphorylation of the insulin receptor tyrosine residues and downstream substrates to reduce the role of insulin [62]. It is well accepted that this enzyme is a promising target for hypoglycemic agent discovery, and a recent review deals with PTP1B inhibitors from natural sources [63]. In the past decade, several BPs with hypoglycemic activity targeting PTB1B have been discovered. For example, BP **2.1** (also **6.1** and **7.1.1**), BP **2.2** (also **6.2**), and BP **2.3** (also **6.3**, **7.1.2**, **7.3.5**, and Scheme 2), isolated from the marine alga *Symphyocladia latiuscula*, showed antidiabetic potential by inhibiting PTP1B, and these compounds also exhibited inhibitory effects on α-glucosidase enzymes, enhancing insulin sensitivity and glucose uptake [64]. In silico molecular docking simulations also revealed the importance of the 7–OH group for H-bond formation and bromine/phenyl ring number for halogen bond interactions with the enzymes [64] (see Section 3). Compound **2.4** (Scheme 2), from the red alga *Rhodomela confervoides*, represents a potential candidate for further development as an antidiabetic agent. Mechanistically, **2.4** competitively inhibited PTP1B via binding to the catalytic site through hydrogen bonds, activated insulin signaling in an insulin-independent manner, and ameliorated insulin resistance through enhancing insulin sensitivity [65,66]. Compound **2.4** also increased glucose uptake in normal and insulin-resistant C2C12 myotubes and HepG2 cells [65,66]. In vivo, long-term oral administration of BP **2.4** could significantly reduce the blood glucose level of streptozotocin-induced diabetic mice without obvious toxic effects [66]. Compound **2.5** (Scheme 2), a synthetic derivative of **2.4** [67], showed significantly enhanced inhibition of PTP1B, and BP **2.5** directly interacted with PTP1B by binding to the enzyme catalytic domain through hydrogen bonds in a competitive mode [68]. BP **2.5** ameliorated the impaired insulin signaling in palmitate-treated C2C12 myocytes. In a db/db mouse diabetic model, BP **2.5** showed hypoglycemic activity and decreased the serum triglycerides and total cholesterol [68]. Further in vivo study confirmed that oral administration of BP **2.5** exhibited a hypoglycemic effect significantly, protecting mice from hyperlipidemia, dyslipidemia, and hyperinsulinemia. In addition, BP **2.5** also enhanced the content of glycogen in the liver and muscles and obviously improved the number of beta cells in the pancreatic islets [68]. BP **2.6** (Scheme 2) with highly chemical structural similarity to compound **2.4** was synthesized. Compared with compound **2.7** (also **1.2** and Scheme 2; IC_50_ = 2.42 µM), compound **2.6** had a relatively higher inhibitory activity on PTP1B (Table 2; IC_50_ = 1.50 µM). Moreover, BP **2.6** could effectively reduce the blood glucose level, total cholesterol, and HbA1c in the C57BL/KSJ-db/db diabetic mouse model [69]. BP **2.8** (also **1.1**, **4.7**, **7.3.3**, and Scheme 2) is a marine natural product isolated from the red alga *Odonthalia corymbifera*, and this compound could inhibit in vitro the activity of both the PTP1B [70] and α-glucosidase (IC_50_ = 0.098 µM) [71]. In vivo, BP **2.8** could efficiently decrease blood glucose, HbA1c, and triglyceride levels, and downregulate the body weight without influencing food and water intake [70]. BP **2.9** (Scheme 2), which was isolated from the red alga *Rhodomela confervoides*, displayed PTP1B inhibition activity with an IC_50_ value of 1.7 µM. Cellularly, BP **2.9** could increase the activity of insulin and could ameliorate palmitic acid-induced insulin resistance in C2C12 myotubes [72]. Moreover, BP **2.9** significantly increased the mRNA expression of carnitine palmitoyl transferase 1B (CPT-1B) and fatty acid binding protein 3 (FABP3) [72], which are closely related to fatty acid oxidation. This suggested that BP **2.9** could improve fatty acid oxidation by inhibiting PTP1B [72].

BP **2.10** (Scheme 2) was a synthetic derivative of the natural BP **2.9**. BP **2.10** also had the function of inhibiting PTP1B (Table 2; IC_50_ = 0.89 µM), and its activity was about twofold higher compared with the lead compound **2.9** (Table 2; IC_50_ = 1.7 µM) [73]. A preliminary SAR study revealed that the tricyclic scaffold and multi-bromine atoms (four to five) attached to the aryl rings were critical for PTP1B inhibition [73]. It has been established that RNA splicing-associated RNA-binding proteins (RBPs) are able to sensitize the insulin signaling pathway, and therefore significantly decrease the blood glucose levels of diabetic BKS db mice [74]. RNA splicing-associated RBPs play a critical role in the posttranscriptional regulation of, e.g., insulin-like growth factor, and their dysregulation usually results in diabetes. BP-mediated modulation of RBPs is a promising therapeutic approach to diabetes. In addition to inhibiting PTP1B, BP **2.10** could also modulate RBPs [75], and the compound was a promising compound to be developed as a novel hypoglycemic agent. When BP **2.9** was hybridized with ribose, a new BP derivative **2.11** (Scheme 2) was synthesized. This compound displayed a very potent and selective inhibitory effect on PTP1B with an IC_50_ value of 199 nM (Table 2) [76].

To find the specific PTP1B inhibitors, a series of BP derivatives were synthesized and evaluated in vitro as PTP1B inhibitors, and **2.12** (also **7.2.2** and Scheme 2) was the strongest one (Table 2; IC_50_ = 0.68 µM), about fourfold more potent than the lead compound BP **2.7**. Further in vivo experiments also revealed that BP **2.12** possessed promising antidiabetic activities [77]. In addition, BP **2.12** displayed high selectivity on PTP1B without any effect on other PTPs, such as TCPTP, LAR, SHP-1, and SHP-2 [77].In order to improve the bioavailability and PTPs selectivity of BPs, a series of new uncharged BP derivatives was synthesized and BP **2.13** (Scheme 2) could effectively bind to PTP1B and inhibit PTP1B with an IC_50_ value of 0.19 ± 0.05 µM (Table 2), and had significant selectivity over other PTPs [78]. BP **2.13** enhanced both the insulin and leptin signaling pathways while it reduced the blood glucose level in diabetic BKS db mice in vivo. Pharmacokinetics study revealed that the compound was absorbed rapidly from the gastrointestinal tract (T_max_ = 0.5 h), and widely distributed in tissues with V_ss_ of 4.25 ± 0.47 L/kg, and t_1/2_ is 0.40 ± 0.03 h. The bioavailability of this compound was 8.31 ± 2.96%. Thus, BP **2.13** could serve as a qualified agent to treat diabetes [78].

Aldose reductase (AR) converts glucose to sorbitol in the polyol pathway and it is an important enzyme to prevent diabetic complications. Some BPs and brominated diarylmethanone derivatives were synthesized and their activity was evaluated against AR [79,80]. Among these derivatives, BP **2.14** (Scheme 2) had the greatest inhibitory effects on AR, with an IC_50_ value of 0.09 µM. Moreover, these derivatives had strong inhibitory effects on α-glucosidase and α-amylase with IC_50_ values of 94.27 nM and 38.11 nM, respectively, stronger than the positive control acarbose [79]. Diarylmetane BP derivatives also possessed excellent inhibitory effect on these metabolic enzymes [80]. Specifically, the synthesized BPs **2.15–2.22** (Scheme 2) could simultaneously inhibit the activity of AR, α-glucosidase, and α-amylase enzymes [80]. The inhibitory potency of BPs **2.15–2.22** against AR varied from 0.129 to 1.30 µM. Among BPs having the same number of hydroxyl groups, BP **2.19** (also **3.37**) displayed the greatest inhibitory effect on AR with an IC_50_ value of 0.129 µM. BPs **2.15–2.22** also exhibited strong inhibitory effect on α-glucosidase, with IC_50_ values from 11.72 to 20.05 nM. Among them, BP **2.18** (also **6.16** and **7.2.18**) possessed the strongest α-glucosidase inhibition ability, while BP **2.17** (also **3.34**, **6.17**, and **7.2.19**) was the lowest. BPs **2.15–2.22** also had strong inhibitory effect on α-amylase, with IC_50_ values from 3.84 to 10.37 nM. Although there was the same number of hydroxyl groups between BP **2.19** and BP **2.21,** their α-amylase inhibition activity was totally different, suggesting that the position of the hydroxyl group plays an important role for their activity [80]. Some newly synthesized BP derivatives **2.23–2.29** (Scheme 2) also inhibited α-glycosidase activity; among them, BP **2.25** (also **3.27** and **6.9**) exhibited the strongest inhibitory effect on α-glycosidase, with an IC_50_ value of 8.73 nM [81], indicating its possibly use for the further development of hypoglycemic agents.

Besides the hypoglycemic activity, some marine BPs have the potential to treat obesity. BP **2.30** (also **3.8**, **4.9**, **5.1**, and Scheme 2), isolated from the red alga *Polysiphonia morrowii*, could inhibit adipogenesis by regulating expression of adipogenic transcription factors and AMP-activated protein kinase activation in 3T3-L1 adipocytes and subsequently inhibited intracellular lipid accumulation and triglyceride level, suggesting the potential to treat obesity [82]. 

From the recent research progress in the antidiabetic activity of BPs, especially their different synthetic derivatives, we can see that more in vivo data has been provided. The major mechanisms for the BPs’ antidiabetic activity have been illustrated systemically and are mainly related to the inhibition of metabolic enzymes. These preclinical in vivo and in vitro trials suggest that BPs may be promising candidates for development of antidiabetic agents. Specifically, it is interesting to investigate whether the inhibition of α-glucosidase and PTP1B by BPs and their derivatives is not only an important role for their anticancer activity but also for their antidiabetic activity.

**Table 2:** Antidiabetic activity and names of compounds in Scheme 2.

No.	IC_50_	Names
**2.1**	7.74 ± 0.14 (a), 2.63 ± 0.11 (b)	2,3,6-tribromo-4,5-dihydroxybenzyl alcohol [64]
**2.2**	8.50 ± 0.45 (a), 7.24 ± 0.02 (b)	2,3,6-tribromo-4,5-dihydroxybenzyl methyl ether [64]
**2.3**	5.29 ± 0.08 (a), 1.92 ± 0.02 (b)	Bis-(2,3,6-tribromo-4,5-dihydroxybenzyl methyl ether) [64]
**2.4**	0.84 (a)	3,4-dibromo-5-(2-bromo-3,4-dihydroxy-6-(ethoxymethyl)benzyl) benzene-1,2-diol [65]
**2.5**	0.63 (a)	3,4-dibromo-5-(2-bromo-3,4-dihydroxy-6-(isopropoxymethyl)benzyl)benzene-1,2-diol [67]
**2.6**	1.50 (a)	3,4-dibromo-5-(2-bromo-3,4-dihydroxy-6-(isobutoxymethyl)benzyl)benzene-1,2-diol [69]
**2.7**	2.42 (a)	2,2′,3,3′-tetrabromo-4,4′,5,5′-tetra-hydroxydiphenyl methane [69]
**2.8**	0.098 (b)	Bis(2, 3-dibromo-4, 5-dihydroxybenzyl)ether [71]
**2.9**	1.7 (a)	3-bromo-4,5-bis(2,3-dibromo-4,5-dihydroxybenzyl)-1,2-benzenediol [72]
**2.10**	0.89 (a)	1-(2-(2,3-dibromo-4,5-dimethoxybenzyl)-4,5-dimethoxybenzyl)-2,3-dibromo-4,5-dimethoxybenzene [73]
**2.11**	0.199 (a)	(2S,3R,4R,5R)-5-(((3-bromo-4,5-dihydroxybenzoyl)oxy)methyl)tetrahydrofuran-2,3,4-triyl tris(3-bromo-4,5-dihydroxybenzoate) [76]
**2.12**	0.68 (a)	5,5’-methylenebis(3,4,6-tribromobenzene-1,2-diol) [77]
**2.13**	0.19 ± 0.05 (a)	3,4-dibromo-5-(5-(4-(4-ethoxyphenoxy)phenyl)oxazol-2-yl)benzene-1,2-diol [78]
**2.14**	94.27 (b), nM 0.09 (c), 38.11 (d), nM	(4-bromo-2,5-dimethoxyphenyl) (phenyl)methanone [79]
**2.15**	15.23 (b), nM, 0.773 (c), nM, 8.03 (d)	(4-bromo-2,5 dihydroxyphenyl) (3,4-dihydroxyphenyl)methanone [80]
**2.16**	19.64 (b), nM, 0.627 (c), nM, 9.12 (d)	4-bromo-2,5 dihydroxyphenyl) (3,4,5-trihydroxyphenyl)methanone [80]
**2.17**	20.05 (b), nM, 0.184 (c), nM, 5.83 (d)	(2-bromo-4-hydroxyphenyl) (4-hydroxyphenyl)methanone [80]
**2.18**	11.72 (b), nM, 0.138 (c), nM, 8.56 (d)	(2-bromo-4-hydroxyphenyl) (phenyl)methanone [80]
**2.19**	12.74 (b), nM, 0.129 (c), nM, 3.84 (d)	4-(2-bromo-4-hydroxybenzyl)benzene-1,2 diol [80]
**2.20**	15.73 (b), nM, 1.30 (c), nM, 6.14 (d)	(2-bromo-4-hydroxyphenyl) (phenyl)methanone [80]
**2.21**	17.52 (b), nM, 0.688 (c), nM, 10.37 (d)	2-bromo-5-(4-hydroxybenzyl)benzene-1,4-diol [80]
**2.22**	17.11 (b), nM, 0.701 (c), nM, 5.16 (d)	2-benzyl-5-bromobenzene-1,4-diol [80]
**2.23**	11.55 (b), nM	(3-bromo-4-methoxyphenyl) (3,4-dimethoxyphenyl)methanone [81]
**2.24**	17.77 (b), nM	(3-bromo-4-methoxyphenyl) (2,3-dibromo-4-hydroxy-5-methoxyphenyl)methanone [81]
**2.25**	8.73 (b), nM	(2,3-dibromo-4-hydroxy-5-methoxyphenyl) (2,5-dibromo-4-methoxyphenyl)methanone [81]
**2.26**	12.62 (b), nM	(3-bromo-4-methoxyphenyl) (2,5-dibromo-4-methoxyphenyl)methanone [81]
**2.27**	26.15 (b), nM	(2,5-dibromo-4-methoxyphenyl) (phenyl)methanone [81]
**2.28**	19.52 (b), nM	(3-bromo-4-hydroxyphenyl) (3,4-dihydroxyphenyl)methanone [81]
**2.29**	24.93 (b), nM	(2,5-dibromo-4-hydroxyphenyl) (phenyl)methanone [81]

Notes: a, PTP1B; b, α-glucosidase; c, aldose reductase; d, α-amylase; unit for IC_50_ is µM, unless labeled as nM.

### 2.3. Antiradical Activity

Free radicals attack macromolecules (e.g., membrane lipids, proteins, enzymes, DNA, and RNA) and play a pivotal role in several health disorders such as cancer, diabetes, neurodegenerative diseases, and inflammatory diseases. Many reports demonstrate that a lot of BPs have potential for antiradical activity, mainly determined by the 1,1-diphenyl-2-picryl hydrazyl (DPPH) and 2,2′-azinobis (3-ethylbenzothiazoline-6-sulfonic acid) diammonium salt (ABTS) radical scavenging methods.

**Scheme 3:**
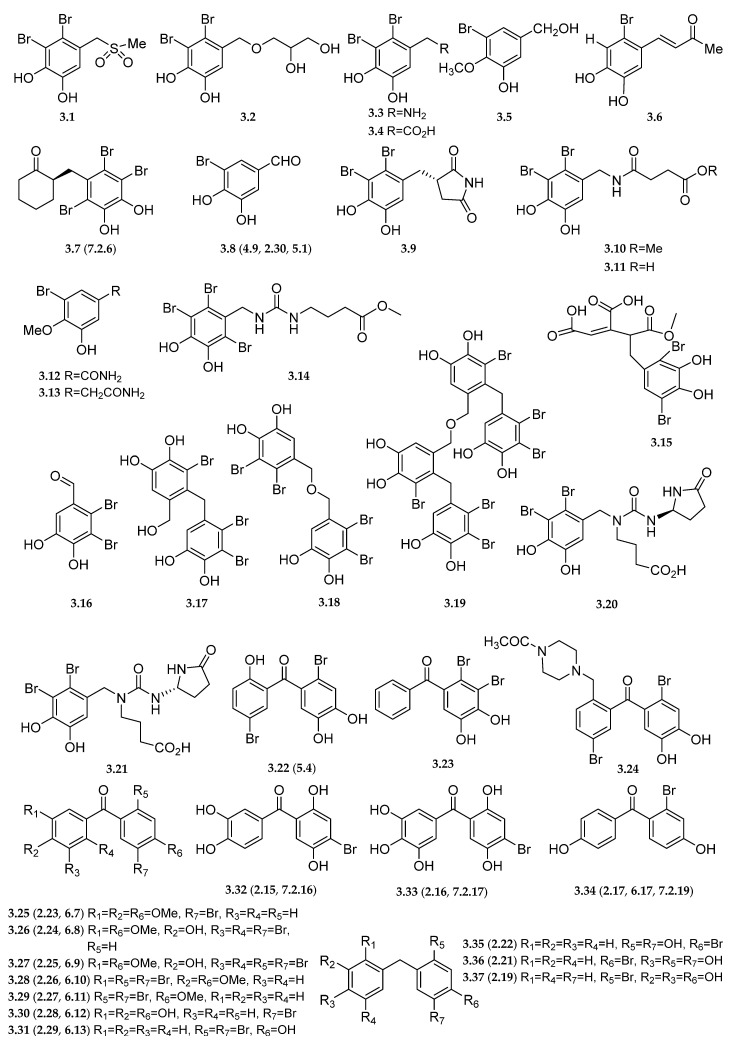
BPs with antiradical activity.

BPs **3.1–3.6** (Scheme 3), isolated from the marine red alga *Rhodomela confervoides*, had the ability to scavenge DPPH and ABTS free radicals, and the IC_50_ values were shown in Table 3 [83]. Recently, a novel BP **3.7** (also **7.2.6** and Scheme 3) was isolated from the red alga *Symphyocladia latiuscula*, and this compound could effectively scavenge DPPH free radicals, with an IC_50_ value of 8.5 μM [84]. BP **3.8** (also **2.30**, **4.9**, **5.1**, and Scheme 3) was found in the marine red algal species *Rhodomela confervoides*, *Polysiphonia morrowii*, and *Polysiphonia urceolata*. Compound **3.8** could scavenge DPPH free radicals, with an IC_50_ value of 20.3 µM [85,86,87]. Further studies showed that BP **3.8** activated ERK- and Akt-mediated Nrf2 signaling cascades and upregulated HO-1 pathway in keratinocytes, and thus induced cytoprotective effects against oxidative stress [88]. Moreover, in addition to scavenge DPPH radicals, BP **3.8** was also reported to scavenge hydroxyl and alkyl radicals. In Vero cells, BP **3.8** could inhibit H_2_O_2_-induced lipid peroxidation, cell death, and apoptosis by inhibiting the production of ROS. Furthermore, compound **3.8** has been shown to significantly inhibit ROS production, lipid peroxidation, and cell death in H_2_O_2_-stimulated oxidative stress in zebrafish embryos [89]. Compound **3.8** also protected human keratinocytes from UVB-induced oxidative stress through removing ROS [90] and inducing the reduced glutathione (GSH) level via the Nrf2-mediated pathway [91]. All these studies confirmed that **3.8** exerted excellent antioxidant activity both in vitro and in vivo.

Some new nitrogen-containing BPs were isolated from the marine red alga *Rhodomela confervoides*, such as BPs **3.9–3.13** (Scheme 3). These BPs showed strong scavenging activity against DPPH free radicals, with IC_50_ values from 5.22 to 23.60 µM, while exhibiting moderate activity against ABTS free radicals, with trolox equivalent antioxidant capacity (TEAC) values from 3.11 to 3.58 mM [92]. Another nitrogen-containing BP **3.14** (Scheme 3) along with **3.15** (Scheme 3), isolated from the marine red alga *Symphyocladia latiuscula*, effectively scavenged DPPH free radicals, with IC_50_ values of 14.5 and 20.5 μg/mL (Table 3) [93]. In one of the few studies using cellular models to evaluate BPs’ antiradical activity, BPs **3.16–3.19** (Scheme 3) were isolated from the red alga *Vertebrata lanosa* and their antiradical activity were investigated, using an oxygen radical absorbance capacity assay, cellular antioxidant activity assay, and cellular lipid peroxidation assay in normal human lung fibroblasts [94]. The results showed that BP **3.17** had stronger antiradical activity when compared with the other three compounds [94].

(R)-rhodomelin A (**3.20**, Scheme 3) isolated from the red alga *Rhodomela confervoides* had potential antiradical activity mainly evaluated by DPPH (IC_50_ = 3.82 µM) and TEAC assays (IC_50_ = 4.37 mM). Similarly, the synthetic (S)-rhodomelin A (**3.21**, Scheme 3) showed powerful inhibition of DPPH radicals with an IC_50_ value of 8.9 μM. Although (S)-rhodomelin A was not so strong as (R)-rhodomelin A (**3.20**), it was more potent than the positive control BHT (IC_50_ = 82.13 μM) [44].

In addition to the natural BPs, many kinds of synthetic BP derivatives have been reported to have good antiradical activities. For instance, 5,2′-dibromo-2,4′,5′-trihydroxydiphenylmethanone (**3.22**, **5.4**, and Scheme 3) and 2,3-dibromo-4,5-dihydroxydiphenylmethanone (**3.23** and Scheme 3), were able to protect human umbilical vein endothelial cells (EC_50_ = 0.4 and 0.8 μM) from H_2_O_2_-induced oxidative stress injury [95]. When a series of nitrogen-containing heterocycles such as piperidine, piperazine, and imidazole replaced the OH group at the 2-position of **3.22**, the products also showed moderate to potent cytoprotective activity against H_2_O_2_-induced injury in EAhy926 cells with **3.24** (Scheme 3; EC_50_ = 0.9 μM) being the most potent compound. A SAR study revealed that the antiradical ability of these analogues strengthened with an increasing number of heterocycles and hydroxyl groups [42]. Furthermore, a molecular docking study demonstrated that compound **3.24** could interact with Keap1, and thus in turn modulate Keap1-Nrf2 protein–protein interaction in order to activate Nrf2-induced downstream protective genes from oxidative stress damage [42] (see Section 3). Some novel synthetic diarylmethanone BPs **3.25–3.31** (Scheme 3) also showed antiradical abilities. These compound could effectively clear DPPH free radicals, with IC_50_ values from 23.10 to 34.65 μg/mL, but they demonstrated a relatively weaker ability to scavenge ABTS radicals, with IC_50_ values from 69.3 to 231 μg/mL [81]. In a recent study, some new BPs **3.32–3.37** (Scheme 3), which had antiradical activities, were synthesized [96]. BPs **3.32–3.37** could effectively scavenge DPPH free radicals with IC_50_ values from 13.32 to 16.44 µg/mL, and ABTS free radicals with IC_50_ values from 5.08 to 7.35 µg/mL. Based on these IC_50_ values, it is concluded that the antiradical capacity of BPs **3.32–3.37** does not vary a lot, indicating that the number of hydroxyl groups and the position of the bromine atom in these compounds have little effect on the antiradical capacity [96].

In the past several decades, the antioxidant activities of BPs are still of interest. The above reported studies further confirm BPs to be one of the potential candidates for the prevention of chronic diseases related to oxidative stress, such as cancer, diabetes, neurodegeneration, and inflammation. Compared with ten years ago, in addition to the natural BPs, more derivatives of BPs have been synthesized and evaluated for their antiradical activity. Moreover, both cellular and animal models have been tested to assess the antiradical capacity of marine BPs, providing more conceivable experimental data.

**Table 3:** Antiradical activity and names of compounds in Scheme 3.

No.	IC_50_/EC_50_	Names
**3.1**	9.52 ± 0.04 (a), 2.06 ± 0.08 (b)	3,4-dibromo-5-((methylsulfonyl)methyl)benzene-1,2-diol [83]
**3.2**	7.43 ± 0.1 (a), 2.11 ± 0.04 (b)	3,4-dibromo-5-((2,3-dihydroxypropoxy)methyl)benzene-1,2-dio [83]
**3.3**	20.47 ± 0.07 (a), 1.87 ± 0.02 (b)	5-(aminomethyl)-3,4-dibromobenzene-1,2-dio [83]
**3.4**	19.84 ± 0.06 (a), 2.87 ± 0.11 (b)	2-(2,3-dibromo-4,5-dihydroxyphenyl)acetic acid [83]
**3.5**	50.58 ± 0.23 (a), 1.60 ± 0.04 (b)	3-bromo-5-(hydroxymethyl)-2-methoxyphenol [83]
**3.6**	8.72 ± 0.05 (a), 3.68 ± 0.12 (b)	(E)-4-(2-bromo-4,5-dihydroxyphenyl)but-3-en-2-one [83]
**3.7**	8.5 (a)	(2R)-2-(2,3,6-tribromo-4,5-dihydroxybenzyl)-cyclohexanone [84]
**3.8**	20.3 (a)	3-bromo-4,5-dihydroxybenzaldehyde [85]
**3.9**	5.22 ± 0.04 (a), 2.87 ± 0.1 (b)	3-(2,3-dibromo-4,5-dihydroxybenzyl)pyrrolidine-2,5-dione [92]
**3.10**	5.70 ± 0.03 (a), 2.14 ± 0.08 (b)	Methyl 4-(2,3-dibromo-4,5-dihydroxybenzylamino)-4-oxobutanoate [92]
**3.11**	5.43 ± 0.02 (a), 2.31 ± 0.11 (b)	4-(2,3-dibromo-4,5-dihydroxybenzylamino)-4-oxobutanoic acid [92]
**3.12**	23.60 ± 0.1 (a), 2.11 ± 0.04 (b)	3-bbromo-5-hydroxy-4-methoxy-benzamide [92]
**3.13**	20.81 ± 0.08 (a), 2.36 ± 0.08 (b)	2-(3-bromo-5-hydroxy-4-methoxyphenyl)acetamide [92]
**3.14**	14.5 (a), µg/mL	Methyl 4-(3-(2,3,6-tribromo-4,5-dihydroxybenzyl)ureido)butanoate [93]
**3.15**	20.5 (a), µg/mL	2-(3-(2,5-dibromo-3,4-dihydroxyphenyl)-1-methoxy-1-oxopropan-2-yl)maleic acid [93]
**3.20**	3.82 ± 0.01 (a), 4.37 ± 0.24 (b)	(R)-Rhodomelin A [44]
**3.21**	8.90 (a)	(S)-Rhodomelin A [44]
**3.22**	0.4 (c)	5,2′-dibromo-2,4′,5′-trihydroxydiphenylmethanone [97]
**3.23**	0.8 (c)	2,3-dibromo-4,5-dihydroxydiphenylmethanone [97]
**3.24**	0.9 (c)	1-(4-(4-bromo-2-(2-bromo-4,5-dihydroxybenzoyl)benzyl)piperazin-1-yl)ethan-1-one [42]
**3.25**	31.50 (a), 198.00 (b), µg/mL	(4-bromo-2,5-dimethoxyphenyl) (phenyl)methanone [81]
**3.26**	28.87 (a), 231.00 (b), µg/mL	(3-bromo-4-methoxyphenyl) (3,4-dimethoxyphenyl)methanone [81]
**3.27**	34.65 (a), 173.25 (b), µg/mL	(3-bromo-4-methoxyphenyl) (2,3-dibromo-4-hydroxy-5-methoxyphenyl)methanone [81]
**3.28**	28.88 (a), 138.6 (b), µg/mL	(2,3-dibromo-4-hydroxy-5-methoxyphenyl) (2,5-dibromo-4-methoxyphenyl)methanone [81]
**3.29**	26.65 (a), 231.00 (b), µg/mL	(3-bromo-4-methoxyphenyl) (2,5-dibromo-4-methoxyphenyl)methanone [81]
**3.30**	23.10 (a), 69.3 (b), µg/mL	(2,5-dibromo-4-methoxyphenyl) (phenyl)methanone [81]
**3.31**	33.00 (a), 115.50 (b), µg/mL	(3-bromo-4-hydroxyphenyl) (3,4-dihydroxyphenyl)methanone [81]
**3.32**	16.44 (a), 6.55 (b), µg/mL	(4-bromo-2,5-dihydroxyphenyl) (3, 4-dihydroxyphenyl)methanone [96]
**3.33**	14.43 (a), 6.86 (b), µg/mL	(4-bromo-2,5-dihydroxyphenyl) (3,4,5-trihydroxyphenyl)methanone [96]
**3.34**	19.24 (a), 7.35 (b), µg/mL	(2-bromo-4-hydroxyphenyl) (4-hydroxyphenyl)methanone [96]
**3.35**	13.32 (a), 6.86 (b), µg/mL	2-benzyl-5-bromobenzene-1,4-diol [96]
**3.36**	13.86 (a), 5.08 (b), µg/mL	2-bromo-5-(4-hydroxybenzyl)benzene-1,4-diol [96]
**3.37**	15.75 (a), 7.71 (b), µg/mL	4-(2-bromo-4-hydroxybenzyl)benzene-1,2-diol [96]

Notes: a, IC_50_ for DPPH inhibition, and unit for IC_50_ is µM, unless labeled as µg/mL; b, IC_50_ for TEAC inhibition and unit for IC_50_ is mM, unless labeled as µg/mL; c, EC_50_ (μM) for H_2_O_2_ inhibition.

### 2.4. Antimicrobial Activity

Microbial infection is still a big challenge worldwide due to drug resistance. One of the major challenges is the limitation of screening libraries [98,99]. Different marine natural products may contribute to improve these chemical libraries, and it has been reported that a lot of BPs possess potent antimicrobial activities. 

**Scheme 4:**
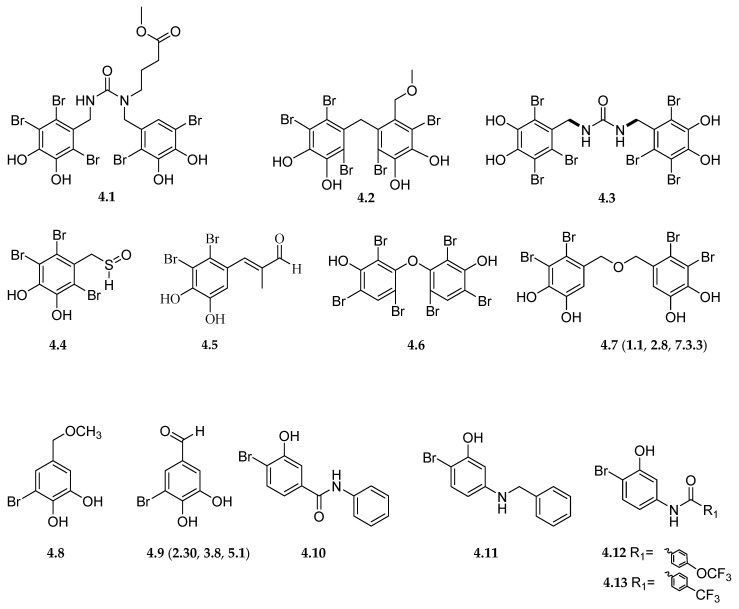
BPs with antimicrobial activity.

Obtained from the marine red alga *Symphyocladia latiuscula*, compounds **4.1**–**4.4** (Scheme 4) revealed antimicrobial activities against *Candida albicans* with the minimum inhibitory concentration (MIC) values in the range of 10 to 37.5 μg/mL (Table 4) [5,6]. *Porphyromonas gingivalis* is an important causative pathogen in human periodontitis, and gingipain R (Rgp) as well as ashemagglutinin A (HgA) proteins play significant roles in the infectious pathway of the pathogen [4]. A methanol extract of the red alga *Kappaphycus*, rich in BPs, showed in vitro antibacterial activities against *P. gingivalis* via inhibiting the gingipain and hemagglutination [4]. BPs were shown to inhibit and control the virulent proteins produced by *P. gingivalis,* thus suggesting their possible applicability in commercial dental products. From the marine red alga *Kappaphycus sp*, the aldehyde **4.5** was isolated (Scheme 4). It exhibited inhibitory activity against both Gram-positive and Gram-negative bacteria [100]. An antimicrobial test by the disc diffusion method showed that BP **4.5** had an obvious inhibitory effect on *Pseudomonas fluorescence* and *Staphylococcus aureus* [100]. Aside from the compounds mentioned above, synthetic BP **4.6** (Scheme 4) showed a favorable antibacterial effect against *Staphylococcus epidermidis* (MIC = 0.556 μM), stronger than the positive control ciprofloxacin [97].

In addition to the human infectious microorganism, some BPs also showed significant effects against phytopathogenic fungi. For example, compound **4.7** (also **1.1**, **2.8**, **7.3.3**, and Scheme 3), isolated from algae *Leathesia nana*, possessed significant antifungal activity against various phytopathogenic fungi, such as *Botrytis cinereal*, *Valsa mali*, and *Fusarium graminearum* [51]. Among these pathogens, *Botrytis cinereal* was the most sensitive to **4.7** (Table 4; IC_50_ = 31 µg/mL). Moreover, **4.7** inhibited the spore germination and the mycelial growth, disrupted the cell membrane, and targeted DNA of *Botrytis cinereal*. This work provided evidence that BPs could be further developed as antifungal agents and applied in the control of phytopathogenic fungi [51].

Moreover, BPs were also reported to have a certain effect against fish virus. From the red alga *Polysiphonia morrowii*, two BPs **4.8–4.9** (Scheme 4) were isolated and characterized [86]. BP **4.8** inhibited fish pathogenic infectious hematopoietic necrosis virus (IHNV) and infectious pancreatic necrosis virus (IPNV) with values of effective concentration for 50% of maximal effect (EC_50_) of 19.04 and 26 μM, respectively, while BP **4.9** (also **2.30**, **3.8**, and **5.1**) only exhibited antiviral activity against IHNV with an EC_50_ of 75 µM (Table 4) [86]. These findings indicated that BPs had potential to be developed as therapeutic agents against fish viral diseases.

A series of *meta*-amido BPs have been designed and synthesized in recent years, which had good inhibitory effects on mycobacterium tuberculosis bacteria and multidrug resistant strains [101]. Compounds **4.10–4.13** (Scheme 4) all showed powerful inhibition against the growth of mycobacterium tuberculosis bacteria H37Ra strain (Table 4; MIC = 0.25–12.5 μg/mL), while they could not inhibit normal Gram-positive and Gram-negative bacteria, indicating their high specificity in the inhibition of tubercle bacillus. Compounds **4.12** and **4.13** also exhibited moderate inhibitory activity against MDR-TB strains of *Mycobacterium tuberculosis*. Furthermore, these BPs showed good metabolic stability in rat livers [101]. Considering the above results, *meta*-amido BPs can become a novel type of anti-tubercular agents.

BPs from marine algae have potential antimicrobial activity that may contribute to the development of antimicrobial dugs against human pathogens, phytopathogenic fungi, and fish virus. More studies are needed to address the toxicity in vivo, the mechanism of the antimicrobial action, and the metabolic stability of marine BPs as antimicrobial agents.

### 2.5. Anti-Inflammatory Activity

**Scheme 5:**
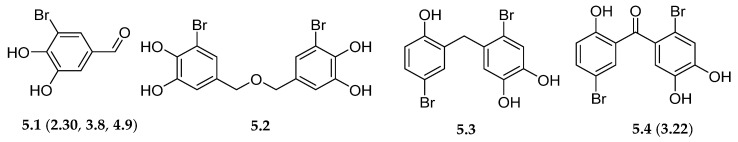
BPs with anti-inflammatory activity.

In the past decade, the anti-inflammatory activities of BPs have attracted great attention. Inflammation is the response triggered by damage to living tissues and has a close relationship to many diseases. Immunoglobulin E (IgE), an important target for atopic dermatitis, induces mast cells to produce inflammatory mediators including various cytokines. Isolated from the red alga *Polysiphonia morrowii*, 3-bromo-4,5-dihydroxybenzaldehyde **5.1** (also **2.30**, **3.8**, **4.9**, and Scheme 5) was reported to alleviate IgE-mediated inflammatory responses in an atopic dermatitis mouse model and RAW 264.7 macrophages, suggesting their therapeutic potential for treating allergic inflammation, e.g., atopic dermatitis [102]. Further studies showed that BP **5.1** reduced the IgE level and inhibited the production of interleukin-6 (IL-6) by downregulating the nuclear factor kappa-light-chain-enhancer of activated B cells (NF-κB) and signal transducer and activator of transcription 1 (STAT1) pathways [102], two major signaling pathways involved in cellular inflammation.

Macrophage activation is associated with diverse pathological processes such as inflammatory disorders, and the activated macrophages yield various inflammatory mediators [7]. Further studies showed that **5.1** reduced CD68^+^ macrophages, M1 and M2 macrophages infiltration, inhibited the phosphorylation of NF-κB, suppressed the secretion of pro-inflammatory cytokines in the injured hearts, and finally, improved cardiac function recovery [103]. This anti-inflammatory activity of **5.1** suppressed the inflammatory factors in myocardial ischemia and reperfusion, and showed myocardial protection via the Akt-PGC1a-Sirt3 pathway [104].

Another BP from the red alga *Polysiphonia morrowii*, bis (3-bromo-4,5-dihydroxybenzyl) ether **5.2** (Scheme 5), significantly decreased lipopolysaccharide (LPS)-induced NO, PGE2, and pro-inflammatory cytokines release via inhibiting the ROS-mediated ERK signaling pathway in RAW 264.7 macrophage cells [7], and thus showed potential in the treatment of inflammatory diseases.

5,2′-dibromo-2,4′,5′-trihydroxydiphenylmethanone **5.3** (Scheme 5), a novel synthetic marine BP derivative, displayed powerful anti-inflammatory effects and could be used in the treatment of acute pyelonephritis (APN) [105]. BP **5.3** could reduce kidney viscera indices and microbial counts in APN rats. Moreover, it inhibited the production of inflammatory mediators, including interleukin-1β (IL-1β) and interleukin-6 (IL-6), and increased the number of CD8^+^ and CD4^+^ T cells, which serve as important inflammatory cytokines and immune cells [105], indicating its further application against renal tissue injury and APN. Another new BP **5.4** (also **3.22**) with anti-inflammatory activity was synthesized. This compound could reduce the expression of inflammatory cytokines, including IL-6, IL-1β,and tumor necrosis factor-α (TNF-α), and could increase the level of anti-inflammatory cytokine IL-10 [106]. BP **5.4** could also reduce the production of nitric oxide and ROS to achieve anti-inflammatory effects. Moreover, BP **5.4** could enhance the phagocytic capacity and reduce LSP-induced inflammation in RAW264.7 cells by activating the PI3K/AKT signaling pathway [106].

### 2.6. Anti-Alzheimer’s Disease (AD) and Parkinson’s Disease (PD) Activity

Neurodegenerative diseases such as AD and PD are major causes of death worldwide and characterized by a progressive loss of specific neuronal cells. Although the discovery of new drugs for these neurodegenerative diseases is still challenging, in recent years, natural BPs have shown protective activities from different neurodegenerative diseases, and therefore are good candidates for the development of new drugs. AD is related to cholinergic deficiency, *β*-amyloid deposition, and formation of tau tangles. Thus, cholinesterases (ChEs, e.g., AChE, BChE), *β*-site amyloid precursor protein cleaving enzyme 1 (BACE1), and glycogen synthase kinase-3*β* (GSK-3*β*) are considered as important targets for anti-AD drugs [9]. PD is another common neurodegenerative disease and ChEs are the major targets for its treatment [107].

**Scheme 6:**
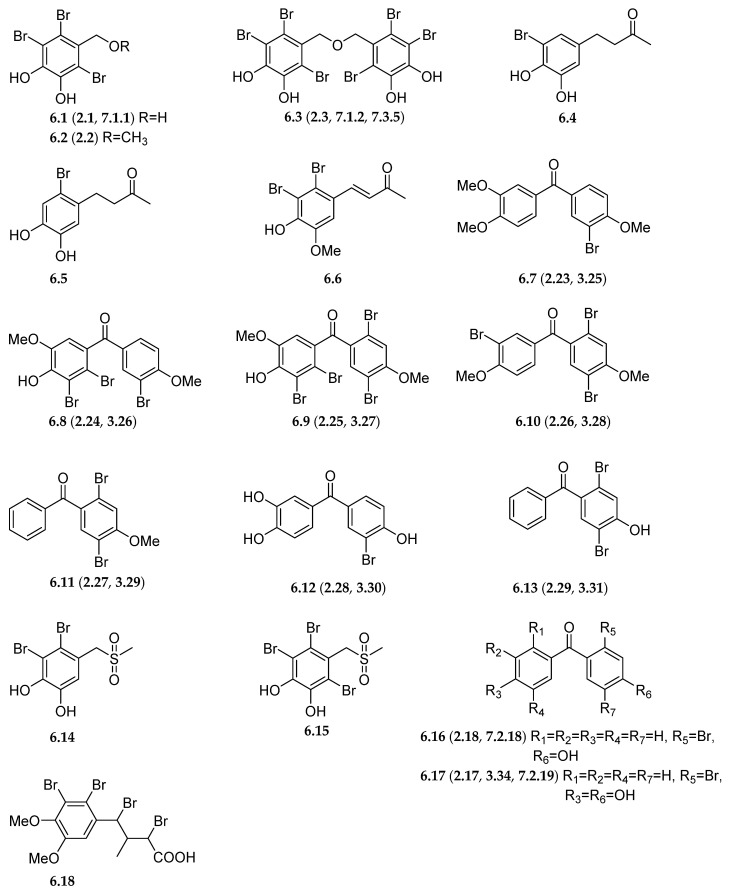
BPs with anti-AD and/or anti-PD activities.

Marine algae have been reported to contain bioactive substances for AD treatment, such as the first marine-derived anti-AD drug sodium oligomannate (GV-971) [108]. Some marine BPs have also shown potential anti-AD activities. BP **6.1** (also **2.1** and **7.1.1**), BP **6.2** (also **2.2**) and BP **6.3** (also **2.3**, **7.1.2**, and **7.3.5**) (Scheme 6), isolated from *Symphyocladia latiuscula* (Harvey) Yamada, had significant effects as potent inhibitors of enzymes, including ChEs, BACE1, and GSK-3*β*. Among them, compound **6.3** exhibited the most potent inhibitory activity against AChE, BChE, and BACE1 (Table 5; IC_50_ values of 2.66, 4.03, and 2.32 μM, respectively). Studies showed that the 7−OH group and bromine number for hydrogen bond and halogen bond interactions seemed important for inhibition of these enzymes [9] (see Section 3). Moreover, BPs **6.1–6.3** exhibited more than 50% inhibition of self-induced A*β*_25–35_ aggregation [9], which may be another mechanism for their anti-AD activity. In addition to anti-AD activity, BPs **6.1–6.3** have also been reported as inhibitors of human monoamine oxidase-A (hMAO-A) [109], which catalyzes the inactivation of multiple neurotransmitters in the treatment of PD. Furthermore, the same research group found that BPs **6.1–6.3** were also good dopamine D_3_/D_4_ receptor agonists. BP **6.3** was the most promising dopamine D_4_ receptor agonist exhibiting EC_50_ value at very low micromolar levels (Table 5) [109]. Since AChE, BChE, BACE1, hMAO-A, and dopaminergic receptors are closely related to the neurodegenerative diseases such as AD and PD, these BPs compounds can possibly be developed as new drugs for the treatment of neurodegenerative disorders. Using PreADMET (v2.0, YONSEI University, Seoul, Korea), ADME predictions of BPs **6.1–6.3** revealed an excellent percentage of plasma protein binding, good intestinal absorption, and high blood−brain barrier penetration [9]. In addition to the BPs mentioned above, BPs **6.4–6.6** (Scheme 6) containing a 4-phenylbutenone moiety, which were isolated from the red alga *Rhodomela confervoides*, also showed ChEs inhibition. BPs **6.4–6.6** inhibited AChE, with *K*_i_ values from 19.02 ± 6.15 to 32.38 ± 8.01 pM. BPs **6.4–6.6** also had the ability to inhibit BChE, with *K*_i_ values from 8.013 ± 3.06 to 13.28 ± 0.07 pM [110]. 

Some synthetic derivatives of the BPs also showed AChE inhibitory activity. For example, BPs **6.7–6.13** (Scheme 6) showed good inhibition of AChE, with *K*_i_ values from 8.94 ± 0.73 to 59.45 ± 14.97 nM. Among them, **6.11** (also **2.27** and **3.29**), which have two bromines in *para*-position and one methoxy, showed the strongest inhibitory ability of AChE, with a *K*_i_ value of 8.94 ± 0.73 nM [81]. In another research study, a series of BP derivatives with a CH_3_SO_2_ group were synthesized, and these compounds had anticholinergic activities [111]. Among these compounds, **6.14** (Scheme 6) inhibited the activity of AChE and BChE, with *K*_i_ values of 1.53 ± 0.23 and 0.93 ± 0.20 nM, respectively, while **6.15** (Scheme 6) inhibited AChE and BChE, with *K*_i_ values of 0.84 ± 0.12 and 3.73 ± 1.03 nM, respectively. Comparing these two compounds, BP **6.14**, with two hydroxyl groups and one bromine group, had stronger activity toward BChE, whereas BP **6.15**, with two hydroxyl groups and three bromine groups, had stronger activity toward AChE [111]. In addition, another two synthetic BPs **6.16** (also **2.18** and **7.2.18**) and **6.17** (also **2.17**, **3.34**, **7.2.19**, and Scheme 6) have shown inhibitory activity against BChE, with IC_50_ values of 22.35 and 30.13 nM, respectively. However, these two compounds had even stronger inhibitory activity against AChE, with IC_50_ values of 10.82 and 13.07 nM, respectively [96]. In another study, a new type of methylated BP **6.18** (Scheme 6) without phenolic hydroxyl groups was synthesized, and exerted an inhibitory effect on AChE activity, with a *K*_i_ value of 159.6 ± 21.9 nM [46]. 

All the above-mentioned results indicate that marine BPs and their derivatives are excellent inhibitors of ChEs; thus, they are candidates for the treatment of neurodegenerative diseases such as AD and PD.

### 2.7. Other Bioactivity

#### 2.7.1. Tyrosinase Inhibitory Activity

**Scheme 7:**
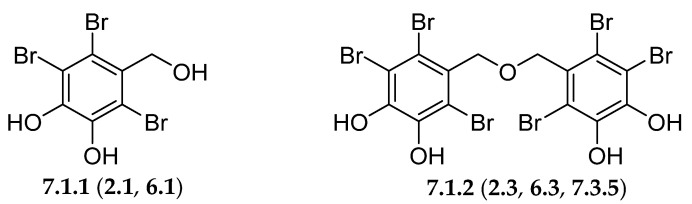
BPs with tyrosinase inhibitory activity.

Tyrosinase is a rate-limiting enzyme for human melanin formation. Therefore, targeting tyrosinase can be used for the development of anti-melanin agents such as depigmenting agents in cosmetology or for the treatment of skin diseases such as hyperpigmentation [112]. BPs **7.1.1** (also **2.1** and **6.1**) and **7.1.2** (also **2.3**, **6.3**, **7.3.5**, and Scheme 7), isolated from *S. latiuscula*, have been shown to inhibit competitively tyrosinase activity, with IC_50_ values of 10.78 ± 0.19 and 2.92 ± 0.04 µM, respectively. A molecular docking study found that catalytic hydrogen bond, hydrogen and halogen interactions contributed to the enzyme inhibition activity (see Section 3). In addition, BPs **7.1.1** and **7.1.2** also showed dose-dependent inhibition of tyrosinase expression levels, melanin content, and intracellular tyrosinase activity in B16F10 melanoma cells [113]. Therefore, the strong tyrosinase inhibitory activity exhibited by these marine BPs isolated from *S. latiuscula,* suggests these algae as a possible source for depigmenting agents used in cosmetology.

#### 2.7.2. Carbonic Anhydrase (CA) Inhibitory Activity

**Scheme 8:**
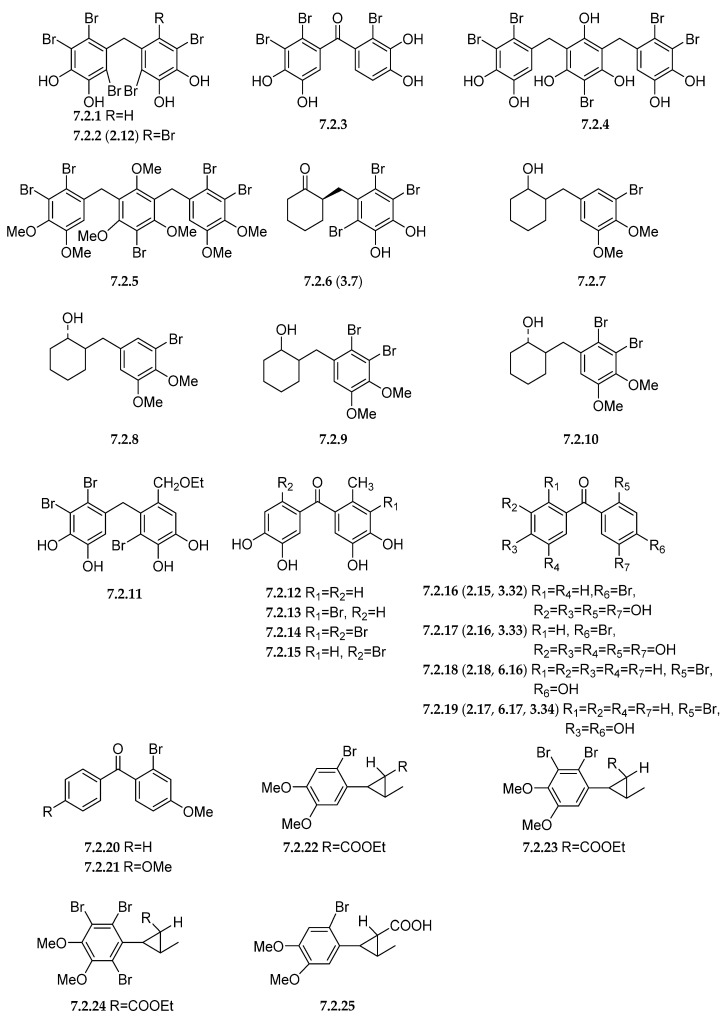
BPs with carbonic anhydrase inhibitory activity.

CA catalyzes the reversible hydration of carbon dioxide into bicarbonate and protons, and it participates in important biological processes in the human body, including acid-base balance, respiration, carbon dioxide, and ion transport. Therefore, inhibiting or activating CA is beneficial to diseases such as edema, glaucoma, obesity, cancer, epilepsy, and osteoporosis [114]. CA inhibition activity is another newly discovered function of marine BPs. For example, BPs **7.2.1** and **7.2.2** (also **2.12** and Scheme 8), obtained from the red alga *Symphyocladia latiuscula*, had weak inhibitory effects on human carbonic anhydrase (hCA) II with IC_50_ values of 86.4 and 38.29 µM, respectively. However, the derivative **7.2.3** (Scheme 8) had a stronger activity (IC_50_ = 0.7 µM) [115]. BP **7.2.4** (Scheme 8) was isolated from the Caribbean red alga *Vidalia obtusaloba*, and had strong inhibitory effects on hCA I, II, IV, and VI, with *K*_i_ values of 12.24, 1.13, 1.84, and 3.41 µM, respectively, whereas BP **7.2.5** (Scheme 8), which was the fully methylated derivative of **7.2.4**, had weaker inhibition against hCA I, II, IV, and VI, with *K*_i_ values of 93.42, 78.49, 57.61, and 45.36 µM, respectively, indicating the importance of the -OH groups in the activity against hCA [116]. BP **7.2.6** (also **3.7** and Scheme 8), isolated from the red alga *Symphyocladia latiuscula* [84], also exhibited good inhibitory activity on hCA I, II, IV, and VI, with *K*_i_ values of 1.67, 0.56, 1.08, and 0.59 µM, respectively [41]. In addition to the natural BPs mentioned above, some synthetic BP derivatives also possess good inhibitory activity on CA. For instance, based on the reduced form of BP **7.2.6**, four O-methylated derivatives **7.2.7–10** (Scheme 8) were synthesized in recent work and showed powerful hCA I, II, IV, and VI inhibitory activity (the *K*_i_ values are given in Table 6). The structures of BPs **7.2.7** and **7.2.9** are *cis*, whereas those of BPs **7.2.8** and **7.2.10** are *trans*. The comparison of these four compounds indicated that the difference in configuration had little effect on the hCA inhibitory activity [41]. Moreover, the natural BP **7.2.11** (Scheme 8) was synthesized and also revealed inhibitory activity for hCA I and II, with *K*_i_ values of 32.7 and 1.26 μM, respectively [39]. Based on BP **7.2.11**, the same research group also synthesized four BPs **7.2.12–15** (Scheme 8) which exhibited enhanced inhibitory activity for hCA I and II, with *K*_i_ values in the range of 13.7–28.5 μM and 0.65–0.92 μM, respectively [39]. Compared with the BPs **7.2.13–15**, **7.2.12** showed better inhibition activity toward hCA I and II, with *K*_i_ values of 13.7 and 0.65 μM, suggesting the attenuation effect of the bromide moiety [39]. When compared with the natural compound BP **7.2.11** with hydrophobic groups, BPs **7.2.12–15** containing hydroxyl groups had stronger inhibition against hCA I and II [39].

Some new BPs **7.2.16–21** (Scheme 8) were synthesized and reported to have hCA I and II inhibitory activity [96]. Among them, BPs **7.2.16–19** inhibited hCA I and II, with IC_50_ values from 6.79 to 8.45 and 5.97 to 9.24 nM, respectively. However, methylated derivatives **7.2.20** and **7.2.21** had similar inhibitory activity on hCA I, with IC_50_ values of 7.61 and 7.51 nM, and on hCA II, with IC_50_ values of 5.58 and 6.07 nM, respectively. The primary SAR indicated that the hydroxyl and methoxy groups had little effect on the hCA inhibitory activity of these compounds. Moreover, among BPs **7.2.16–21** there was no significant difference in hCA I and II inhibition, indicating that they were non-specific hCA inhibitors [96].

In another study, methylated BPs **7.2.22–24** (Scheme 8) were synthesized and exhibited excellent hCA I inhibition profile with *K*_i_ values of 8.4 ± 2.3, 10.7 ± 2.9, and 7.8 ± 0.9 nM, respectively [46]. In addition to hCA I, BP **7.2.22** together with **7.2.25** (Scheme 8), also had strong inhibition activity on hCA II, with *K*_i_ values of 48.3 ± 1.3 and 43.1 ± 1.7 nM, respectively [46]. In general, both the natural BPs and their synthesized derivatives may be candidates as potent CA inhibitors, and thus protect the body from a series of diseases such as edema, glaucoma, obesity, cancer, epilepsy, and osteoporosis.

#### 2.7.3. Glucose 6-Phosphate Dehydrogenase (G6PD) Inhibitory Activity

**Scheme 9:**
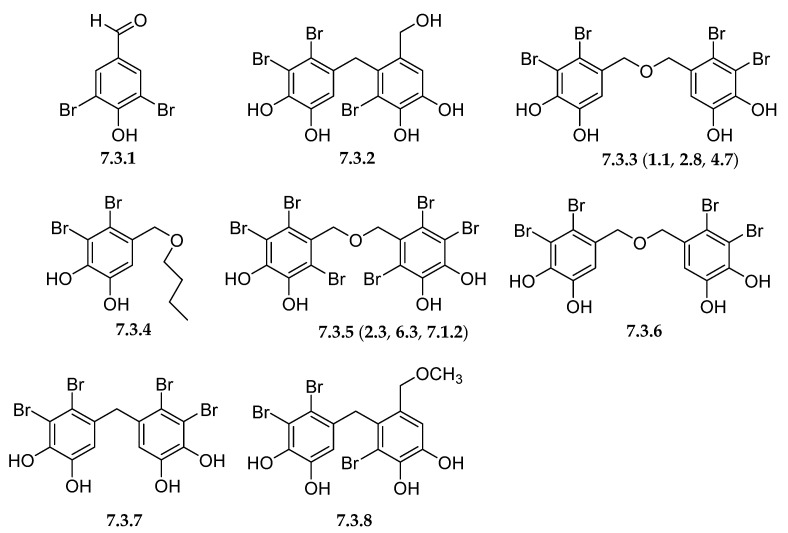
BPs with glucose 6-phosphate dehydrogenase inhibitory activity.

G6PD is a key enzyme in the pentose phosphate pathway, and a potential target for the treatment of obesity and cancer. Some BPs exhibited inhibitory activity on G6PD. For example, BPs **7.3.1**–**7.3.3** (Scheme 9) isolated from three rhodomelaceae algae species (*Laurencia nipponica*, *Polysiphonia morrowii*, and *Odonthalia corymbifera*) had inhibitory effects on G6PD with IC_50_ values ranged from 0.85 ± 0.1 to 76.6 ± 0.1 µM (Table 7). Among them, BP **7.3.3** (also **1.1**, **2.8**, and **4.7**) had the strongest inhibitory effect on G6PD with an IC_50_ value of 0.85 ± 0.1 µM (Table 7). Primary SAR studies found that dimer BPs exhibited stronger inhibitory effects than the monomers, and as the substitution of bromine atoms increased, the inhibitory effects also increased [117]. The **7.3.4** (Scheme 9) was a novel BP isolated from the algae *Odonthalia corymbifera*, *Neorhodomela aculeata*, and *Symphyocladia latiuscula*. This compound had an inhibition activity on G6PD of prokaryotic *Leuconostoc mesenteroides*, with an IC_50_ value of 321 ± 18 µM (Table 7). However, the inhibitory effect of BP **7.3.4** on G6PD of another eukaryotic *Saccharomyces cerevisiae* was not obvious. This specificity was presumed to be due to the difference of the recognition site of the hydrophobic alkyl group on the side chain of the compound [118]. In addition, other BPs were used in this study to investigate the effect of compound structure on inhibition of G6PD derived from prokaryotes and eukaryotes. Studies found that **7.3.5** (also **2.3**, **6.3**, and **7.1.2**) and **7.3.6** (Scheme 9) had a stronger inhibitory effect on G6PD of prokaryotic *Leuconostoc mesenteroides*, with IC_50_ values of 0.97 ± 0.1 and 0.85 ± 0.1 µM, respectively (Table 7), but BPs **7.3.7**, **7.3.8**, and **7.3.2** (Scheme 9) had stronger inhibitory effects on G6PD of eukaryotic *Saccharomyces cerevisiae* with IC_50_ values of 0.47 ± 0.03, 0.53 ± 0.18, and 0.39 ± 0.23 µM, respectively (Table 7). It can be seen that the dibenzyl ether type symmetric BP dimers have strong inhibitory effects on G6PD of prokaryotic *Leuconostoc mesenteroides*, while the diarylmethane type symmetric BP dimers have strong inhibitory effects on G6PD of eukaryotic *Saccharomyces cerevisiae* [118].

#### 2.7.4. Possible Toxicological Effects of BPs

**Scheme 10:**
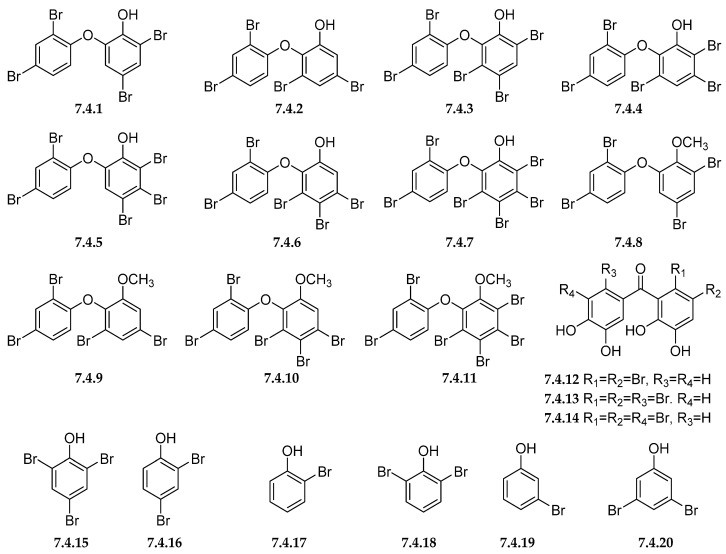
BPs with possible toxicological effects.

Not all the natural BPs and their derivatives show beneficial health effects. Some BPs are suspected to have negative impacts on human and animal health as well as on the environment. In addition to the toxic effects that we reviewed in 2011 [38], some novel negative effects of BPs have been reported. For example, seven hydroxylated polybrominated diphenyl ethers (OH-PBDEs, **7.4.1**–**7**; Scheme 10) and four methoxylated polybrominated diphenyl ethers (MeO-PBDEs, **7.4.8**–**11**; Scheme 10) were discovered in macroalgae and blue mussels [28]. According to the reports, PBDEs had the potential for endocrine disorders, neurological toxicities, and genotoxicity [119,120]. Moreover, they also caused environmental pollution and were difficult to degrade [121]. Fortunately, PBDEs could be degraded by UV irradiation [122].

Three BPs **7.4.12**–**14** (Scheme 10) have been reported to affect the activity of paraoxona (arylesterase, EC 3.1.8.1, PON1) [40]. PON1 is an important enzyme preventing atherosclerosis. Moreover, it also has anti-atherogenic and antioxidant effects, and hydrolyzes many chemical agents [40]. Compounds **7.4.12**–**14** exhibited moderate inhibition of PON1 with IC_50_ values of 0.832, 1.11, and 0.701 mM, respectively, and the inhibition of PON1 led to the increase of oxidative stress and oxidized low density lipoproteins, which enhances the risk of cardiovascular disease [40]. Therefore, the possible toxicity of BPs for all life forms and environment should be kept in mind.

UDP-glucuronosyltransferases (UGTs), an important member of phase II drug-metabolizing enzymes, have been demonstrated to play an important role in the elimination of various substances through conjugation of lipophilic substances with glucuronic acids. UGTs-catalyzed metabolic reaction can decrease the activity and increase the water solubility of toxic compounds. Therefore, inhibition of these UGTs will slow down the excretion of xenobiotics [123]. A recent study found that BPs **7.4.15**–**20** (Scheme 10) had inhibitory effects on UGTs, with UGT1A7 being the most sensitive UGT isoform. BP **7.4.15** has shown in vitro inhibitory effects on UGT1A3, UGT1A7, and UGT2B7, with *K*_i_ values of 2.85, 3.99, and 31.00 µM, respectively [124]. The SAR of BPs showed that the position and number of bromine atom played a vital role for the inhibition activity. In addition, the hydrophobic contacts of BPs with the active cavity of UGTs also contributed to the UGTs inhibition. The in vitro-in vivo extrapolation also demonstrated that BP **7.4.15** had moderate inhibitory activities in vivo on UGT1A3 and UGT1A7 [124]. Therefore, this BP may have a negative effect on the human body by targeting UTGs and affecting the pharmacokinetics of other agents.

## 3. Mode of Interaction between the BP Compounds and Targets

Understanding the mode of interaction is important in order to modify natural BPs into something even more efficient. BPs can interact in typical three ways: hydrogen bonding, halogen bonding, π–π interactions involving aromatic rings, and hydrophobic interactions. In the latter case, the polysubstituted benzene ring, OH, and/or Br substituents will act as an electron deficient entity. Hydrogen bonding is well understood by now [125]. The importance of halogen bonding in the interaction of small molecules with proteins has become more and more clear. The halogen is typically interacting with an electron-rich site (Lewis base), usually in a linear fashion. The donor and the acceptor should be within the sum of the van der Waals radii. The acceptor is typically a C=O group from the protein backbone, but another oxygen is also at play, and so are N- and S-containing groups. Interaction with π-systems is also possible, whereas interactions with C-H bonds are secondary interactions [126].

**Figure 1:**
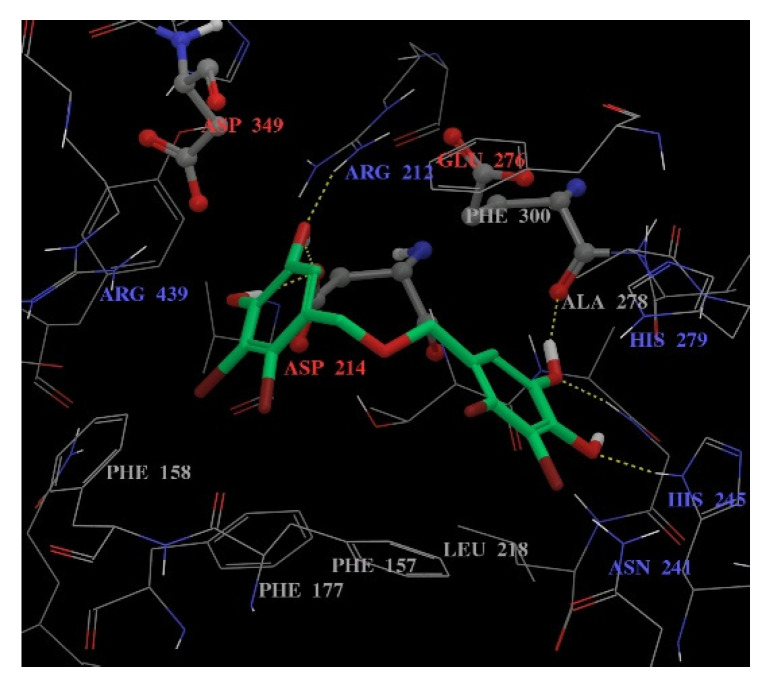
Molecular docking of **2.8** to the active site of α-glucosidase [71].

Docking of BP **2.8** (also **1.1**, **4.7**, and **7.3.3**) into α-glucosidase has been studied (Figure 1). The docking results indicated the interaction between BP **2.8** and α-glucosidase was driven by both hydrophobic forces and hydrogen bonds. The docked BP **2.8** molecule was completely buried in the α-glucosidase binding pocket with part of the molecule reaching the catalytic center and overlapping with the position of glucose, and the rest of the molecule extending toward the protein surface [71].

**Figure 2:**
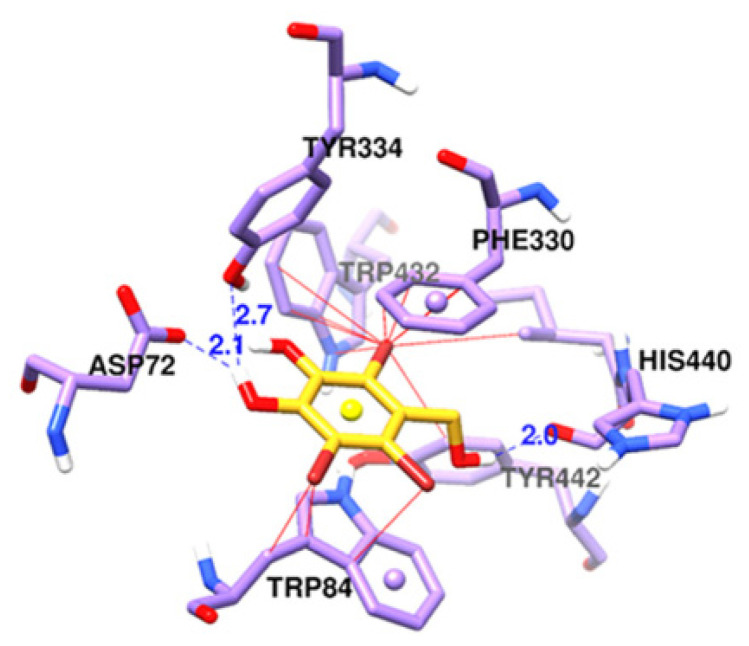
Molecular docking of **2.1** to the active site of *Tetronarce californica* AChE [9]. (https://pubs.acs.org/doi/10.1021/acsomega.9b01557) Reproduced with permission from the American Chemical Society. Readers who wish to obtain further permissions related to the material excerpted should be directed to the ACS.

**Figure 3:**
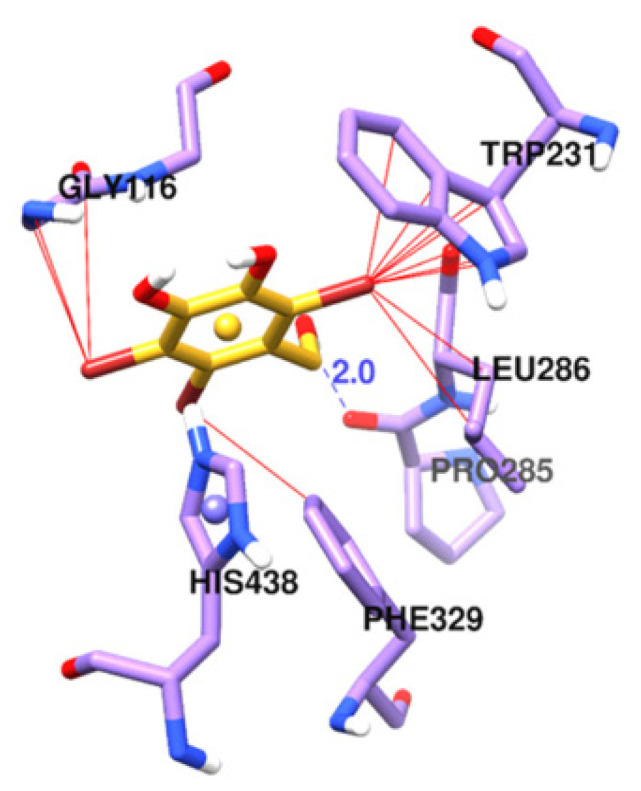
Molecular docking of **2.1** to the active site of human BChE [9]. (https://pubs.acs.org/doi/10.1021/acsomega.9b01557) Reproduced with permission from the American Chemical Society. Readers who wish to obtain further permissions related to the material excerpted should be directed to the ACS.

**Figure 4:**
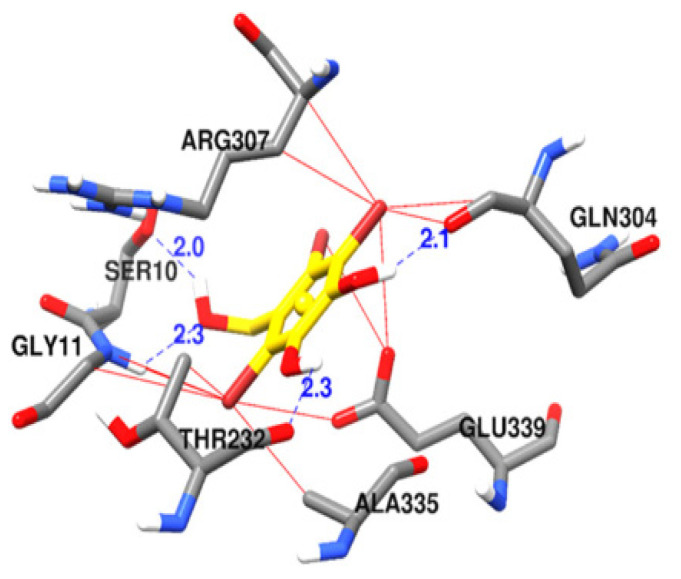
Molecular docking of **2.1** to the active site of human BACE1 [9]. (https://pubs.acs.org/doi/10.1021/acsomega.9b01557) Reproduced with permission from the American Chemical Society. Readers who wish to obtain further permissions related to the material excerpted should be directed to the ACS.

Docking of BP **2.1** (also **6.1** and **7.1.1**), BP **2.2** (also **6.2**), and BP **2.3** (also **6.3**, **7.1.2**, and **7.3.5**) into AChE has been studied [9]. From Figure 2, it can be seen that most of the interactions involving bromine occur with aromatics, Trp84 and Phe330. For **2.2**, an interaction between bromine is to a C=O, again to Phe330, and to the OH bond of Ser122. For **2.3**, an interaction is seen between bromine and a NH of Gly11, and again to the aromatic ring of Trp84. Strangely enough, the interactions with the aromatic rings are shown at the rims of the rings, whereas other studies show binding to the center [127]. Docking of **2.1**–**2.3** has also been done with BChE (Figure 3) and with BACE1 (Figure 4) [9].

Docking of BP **2.1** (also **6.1** and **7.1.1**), BP **2.2** (also **6.2**), and BP **2.3** (also **6.3**, **7.1.2**, and **7.3.5**) has also been done with the PTP1B enzyme. For **2.1**, the bromine atoms were involved in interactions with Thr263, Val184, Gln266, Gly183, Arg221, Phe1823, Lys116, and Trp176 [64]. An inspection of the graphics revealed major interactions to a side-chain nitrogen of Arg221 and to the OH group of Thr263, whereas the remaining interactions are to CH bonds. Docking of **2.1–2.3** has also been done to the oxy-form of *Agaricus bisporus* tyrosinase [113]. The interactions are not specified.

In another docking study, (2S)-2-amino-3-(3-bromo-5-hydroxy-4-methoxyphenyl) propanoic acid was docked to *Porphyromonas gingivalis* Peptidyl. In this case, the bromine points toward a lysine. Other docking experiments bind 2-N-(2,3-dibromo-4,5-dihydroxybenzyl)-9-β-α-ribofuranosyloxyguanosine with Rgp or HgA [9]. In those two cases, the bromine does not seem to play a role in the binding. The binding of **3.24** to Keap1-Kelch was studied [42]. One bromine is pointing toward the hydrophobic region, but the bromines do not seem to play a major role in binding. The authors of that study found that the alkyl substituents are much more important, which is not surprising as the BP is only a minor part of the molecule. Docking of **2.11** has also been done, but with no emphasis on bromine [76]. It would be interesting with a more detailed analysis of the docking experiments following the principles mentioned above. SAR experiments showed in two cases increased binding with an increasing number of bromines [73,117], whereas no effect was found in a third study [46].

**Figure 5:**
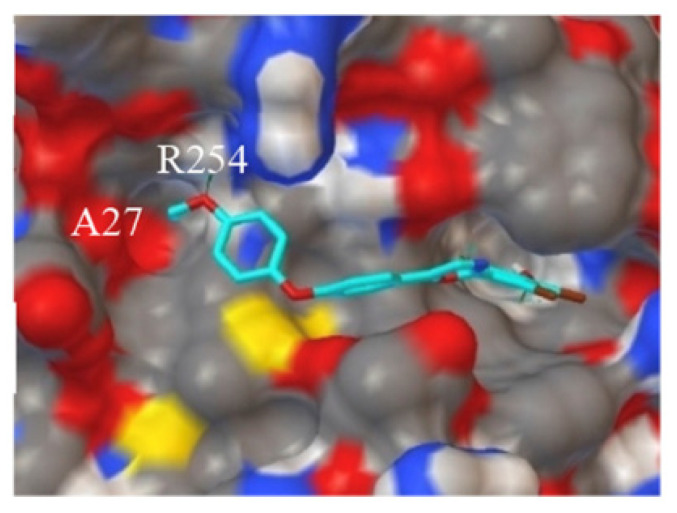
Molecular docking of **2.13** to the active site of PTP1B [78]. (https://doi.org/10.1016/j.ejmech.2019.01.057) Reproduced with permission from the Elsevier. Readers who wish to obtain further permissions related to the material excerpted should be directed to the Elsevier.

Docking of BP **2.13** into PTP1B has been studied and revealed that hydrogen bonding and hydrophobic interactions are the major action modes between BP **2.13** and PTP1B. As can be seen from Figure 5, the ethoxy group of BP **2.13** at the *para*-position of the benzene ring can occupy the hydrophobic cavity near Ala27, which forms selective inhibition of PTP1B. Moreover, the ethoxy group can form hydrogen bond with Arg254 in PTP1B [78].

**Figure 6:**
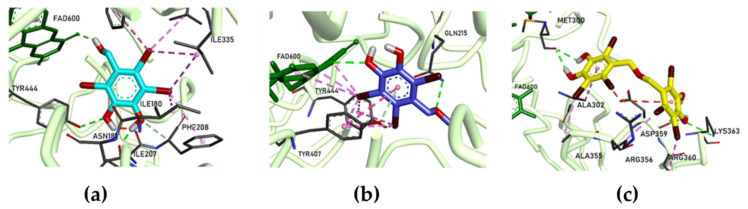
Molecular docking of **6.1** (**a**), **6.2** (**b**), and **6.3** (**c**) to the active site of human hMAO-A [109]. (https://pubs.acs.org/doi/10.1021/acs.jafc.0c00007) Reproduced with permission from the American Chemical Society. Readers who wish to obtain further permissions related to the material excerpted should be directed to the ACS.

Docking of BP **6.1** (also **2.1** and **7.1.1**), BP **6.2** (also **2.2**), and BP **6.3** (also **2.3**, **7.1.2**, and **7.3.5**) into hMAO-A and dopamine D_3_/D_4_ receptor has been studied. BPs **6.1**–**6.3** competitively binds to the catalytic site of hMAO-A. As can be seen from Figure 6, phenolic hydroxyl groups in BP **6.1** can form hydrogen bonds with Tyr444, FAD600, Asn181, and Ile207. In addition, methoxyl and phenolic hydroxyl groups in BP **6.2** can form hydrogen bonds with Gln215 and FAD600, while phenolic hydroxyl groups in BP **6.3** can form hydrogen bonds with Lys363, Met300, and Asp359 [109].

**Figure 7:**
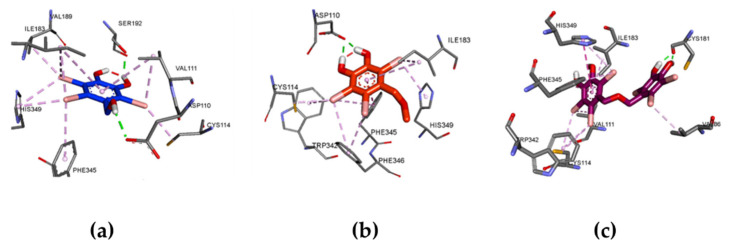
Molecular docking of **6.1** (**a**), **6.2** (**b**), and **6.3** (**c**) to the active site of D_3_R [109]. (https://pubs.acs.org/doi/10.1021/acs.jafc.0c00007) Reproduced with permission from the American Chemical Society. Readers who wish to obtain further permissions related to the material excerpted should be directed to the ACS.

**Figure 8:**
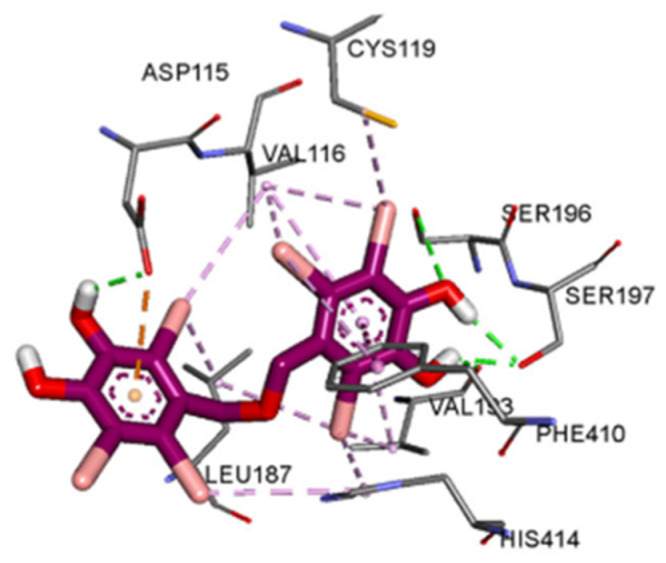
Molecular docking of **6.3** to the active site of D_4_R [109]. (https://pubs.acs.org/doi/10.1021/acs.jafc.0c00007) Reproduced with permission from the American Chemical Society. Readers who wish to obtain further permissions related to the material excerpted should be directed to the ACS.

BP **6.1** (also **2.1** and **7.1.1**), BP **6.2** (also **2.2**), and BP **6.3** (also **2.3**, **7.1.2**, and **7.3.5**) docked to D_3_R ligands indicated that Asp110, Cys114, His349, and Phe345 are the most important residues, and hydrogen bonds are formed between the compound and these amino acid residues (Figure 7). Moreover, BPs **6.1**–**6.3** were also docked to D_4_R, and BP **6.3** showed the strongest combination ability to D_4_R. Phenolic hydroxyl groups in BP **6.3** can form various hydrogen bonds with Ser196, Asp115, Val193, and Ser197 residues (Figure 8) [109].

**Figure 9:**
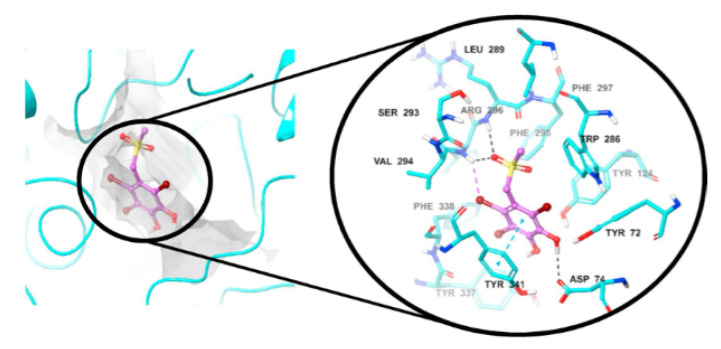
Molecular docking of **6.15** to the active site of human AChE [111]. (https://doi.org/10.1016/j.bioorg.2018.12.012) Reproduced with permission from the Elsevier. Readers who wish to obtain further permissions related to the material excerpted should be directed to the Elsevier.

**Figure 10:**
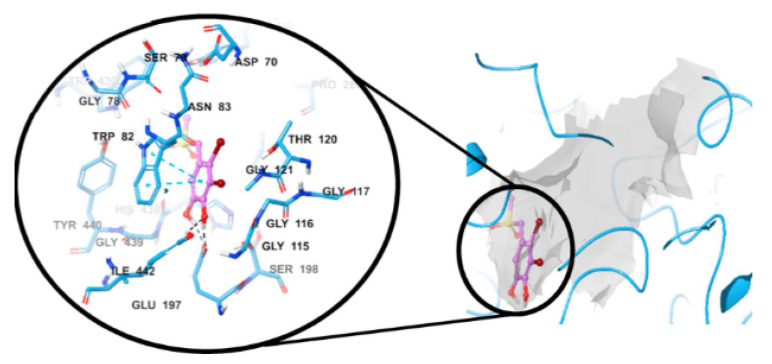
Molecular docking of **6.14** to the active site of human BChE [111]. (https://doi.org/10.1016/j.bioorg.2018.12.012) Reproduced with permission from the Elsevier. Readers who wish to obtain further permissions related to the material excerpted should be directed to the Elsevier.

Docking of BP **6.14** into BChE and **6.15** into AChE has been studied and hydrophobic forces are involved in these interactions. As shown in Figure 9, almost all 3,4,6-tribromo benzene-1,2-diol and methanesulfonyl moieties of BP **6.15** are surrounded by hydrophobic residues in the AChE catalytically active site, including Tyr72, Tyr124, Trp286, Leu289, Val294, Phe295, Phe297, Tyr337, Phe338, and Tyr341. As shown in Figure 10, the 3,4-dibromo benzene-1,2-diol portion of BP **6.14** is surrounded by hydrophilic residues Glu197 and Ser198 and hydrophobic residue Trp82, but the CH_3_SO_2_ portion is surrounded by the hydrophobic residues Met437 and Tyr440 of BChE [111].

## 4. Conclusions

In the last decade, intensive efforts and great progress have been made in the isolation, synthesis, and novel biological activities by screening of BPs. The marine BPs and the synthetic derivatives continue to enlarge the chemical library and improve the opportunity to discover new agents, while interesting novel BPs will still be found, and derivatives will be designed and synthesized in the future. The pharmacology activities of BPs and their derivatives are mainly focused on anticancer, antidiabetic, anti-obesity, antiradical, antimicrobial, anti-inflammatory, anti-AD, anti-PD, and enzyme inhibitory activities. SAR studies on the natural and synthetic BPs reveal that some core structure or substituents may play a critical role for the biological activity. However, it has been proved difficult to identify a selective, safe, and effective new drug from these marine-derived BPs. Due to the limited amount of natural BPs in marine algae, immediate in vivo investigations may hinder the research and development of BP-based drugs. However, the number of semisynthetic and synthetic brominated compounds has been increasing, and more in vivo trials have been recently conducted than were conducted ten years ago. In addition, some mechanisms underlying the BPs’ distribution and mechanisms of their targets are being illustrated, which will enhance the structure optimization of the natural BPs. The discovery of natural marine BPs with novel skeletons, the screening of their bioactivities, their structure modification via synthetic and semisynthetic routes, and the optimization of pharmacokinetic and pharmacodynamics parameters continue to be crucial subjects in the future investigations of this area in order to provide new lead compounds for drug development.

## Figures and Tables

**Table 1:** Anticancer activity and names of compounds in Scheme 1.

No.	IC_50_ (μM) and Cells	Names
**1.1**	13.9 (K562), µg/mL	Bis(2,3-dibromo-4,5-dihydroxybenzyl)ether [50]
**1.2**	17.63 (Hela), 11.37 (RKO), 10.58 (HCT-116), 23.69 (U87), 8.7 (Bel7402), µg/mL, 3.6 (Human umbilical vein endothelial cells)	Bis-(2,3-dibromo-4,5-dihydroxy-phenyl)-methane [53]
**1.3**	1.4 (K562), 4.8 (HeLa), 1.9 (MCF-7), 5.5 (MDB-MB-231), µg/mL	(1′R,5′S,6′S)-2-(3′,5′-dibromo-1′,6′-dihydroxy-4′-oxocyclohex-2′-enyl)acetonitrile [54]
**1.4**	6.6 ± 0.82 (A549), 9.2 ± 0.84 (Bel7402), 13.2 ± 2.42 (HepG2), 9.1 ± 0.13 (HCT-116), 7.4 ± 0.22 (HeLa), µg/mL	(E)-3-(3-bromo-4,5-dimethoxybenzylidene-N-(4-bromophenyl)-2-oxoindoline-5-sulfonamide [55]
**1.5**	14.4 ± 1.86 (A549), 12.3 ± 0.23 (Bel7402), 14.3 ± 0.86 (HepG2), 9.8 ± 0.55 (HCT-116), 8.3 ± 0.67 (HeLa), µg/mL	(E)-N-(4-bromophenyl)-3-(2,3-dibromo-4,5-dimethoxybenzylidene)-2-oxoindoline-5-sulfonamide [55]
**1.6**	10.1 ± 0.72 (A549), 9.7 ± 2.35 (Bel7402), 11.2 ± 1.26 (HepG2), 8.6 ± 0.26 (HCT-116), 18 ± 0.13 (HeLa), µg/mL	(E)-3-(2,3-dibromo-4,5-dimethoxybenzylidene)-5-(morpholinosulfonyl)indolin-2-one [55]
**1.7**	12.5 ± 0.19 (A549), 7.9 ± 0.26 (Bel7402), 25 ± 0.18 (HepG2), 6.1 ± 0.23 (HCT-116), 8.6 ± 0.14 (HeLa), µg/mL	(E)-N-(adamantan-1-yl)-3-(3-bromo-4,5-dimethoxybenzylidene)-2-oxoindoline-5-sulfonamide [55]
**1.8**	12.5 ± 0.45 (A549), 12.5 ± 0.39 (Bel7402), 14.2 ± 0.77 (HepG2), 8.2 ± 0.54 (HCT-116), 9.3 ± 0.47 (HeLa), µg/mL	(E)-N-(adamantan-1-yl)-3-(2,3-dibromo-4,5-dimethoxybenzylidene)-2-oxoindoline-5-sulfonamide [55]
**1.9**	3.15 ± 0.43 (A549), 6.10 ± 0.78 (HepG2), 4.42 ± 0.72 (Bel7402), 5.74 ± 0.26 (HCT-116), 4.23 ± 0.32 (Caco2), µg/mL	(E)-3-(3-bromo-5-methoxy-4-(2-(piperidin-1-yl)ethoxy)benzylidene)-N-(4-bromophenyl)-2-oxoindoline-5-sulfonamide [56]
**1.10**	4.29 ± 0.79 (A549)	3-(4-(3-([1,4′-bipiperidin]-1′-yl)propoxy)-3-bromo-5-methoxybenzylidene)-N-(4-bromophenyl)-2-oxoindoline-5-sulfonamide [57]
**1.11**	4.78 ± 0.56 (A549), 9.99 ± 1.81 (95D), 6.14 ± 0.60 (NCI-H460), µg/mL	3-(3-bromo-5-methoxy-4-(3-(piperidin-1-yl)propoxy)benzylidene)-N-(4-bromophenyl)-2-oxoindoline-5-sulfonamide [58]
**1.12**	2.39 ± 0.43 (SK-OV-3), 5.45 ± 1.03 (Bel7402), 4.60 ± 0.38 (HepG2)	2-(2,3-dibromo-4,5-dimethoxybenzylidene)hydrazine-1-carbo-thioamide [59]
**1.13**	1.89 ± 0.22 (HCC-1937)	(E)-4-(2-(2,3-dibromo-4,5-dimethoxybenzylidene)hydrazine-1-carbothioamido)benzoate [60]

Notes: unit for IC_50_ is μM, unless labeled as μg/mL.

**Table 4:** Antimicrobial activity and names of compounds in Scheme 4.

No.	MIC/IC_50_/EC_50_ and Microbe	Names
**4.1**	MIC 25 µg/mL (a)	Methyl 4-{(2,5-dibromo-3,4-dihydroxybenzyl) [(2,3,6-tribromo-4,5-dihydroxybenzyl)carbamoyl]amino}butanoate [5]
**4.2**	MIC 12.5 µg/mL (a)	2,5-dibromo-3,4-dihydroxy-6-(2,3,6-tribromo-4,5-dihydroxybenzyl)benzyl methyl ether [5]
**4.3**	MIC 10 µg/mL (a)	symphyocladin G [6]
**4.4**	MIC 37.5 µg/mL (a)	2,3,6-tribromo-4,5-dihydroxybenzyl methyl sulphoxide [6]
**4.6**	MIC 0.556 µg/mL (b)	2,4,6,2′,4′,6′-Hexabromodiorcinol [97]
**4.7**	IC_50_ 31 µg/mL (c)	Bis-(2,3-dibromo-4,5-dihydroxybenzyl)-ether [51]
**4.8**	EC_50_ 19.04 µM (IHNV) EC_50_ 26 µM (IPNV)	2, 3-bromo-4,5-dihydroxybenzyl methyl ether [86]
**4.9**	EC_50_ 75 µM (IHNV) EC_50_ > 100 µM (IPNV)	3-bromo-4,5-dihydroxybenzaldehyde [86]
**4.10**	MIC 12.5 µg/mL (d)	4-bromo-3-hydroxy-N-phenylbenzamide [101]
**4.11**	MIC 12.5 µg/mL (d)	5-(benzylamino)-2-bromophenol [101]
**4.12**	MIC 0.5 µg/mL (d)	N-(4-bromo-3-hydroxyphenyl)-4-(trif luoromethoxy)-benzamide [101]
**4.13**	MIC 0.25 µg/mL (d)	N-(4-bromo-3-hydroxyphenyl)-4-(trif luoromethyl)-benzamide [101]

Notes: a, *Candida albicans* (ATCC 10231); b, *Staphylococcus epidermidis*; c, *Botrytis cinereal*; d, *Mycobacterium tuberculosis* H37Ra.

**Table 5:** Anti-AD and anti-PD activities and names of compounds in Scheme 6.

No.	IC_50_/*K*_i_/EC_50_	Names
**6.1**	7.31 ± 0.25 (a), 8.95 ± 2.18 (b), 5.16 ± 0.60 (c), 229.42 ± 12.05 (d), 204.94 ± 4.46 (e)	2,3,6-tribromo-4,5-dihydroxybenzylalcohol [9,109]
**6.2**	9.61 ± 0.35 (a), 14.41 ± 0.27 (b), 4.79 ± 0.82 (c), 140.01 ± 15.08 (d), 63.16 ± 0.4 (e)	2,3,6-tribromo-4,5-dihydroxybenzyl methyl ether [9,109]
**6.3**	2.66 ± 0.24 (a), 4.03 ± 0.15 (b), 2.32 ± 0.10 (c), 56.46 ± 2.48 (d), 89.31 ± 2.45 (e), 18.72 ± 2.80 (f)	Bis(2,3,6-tribromo-4,5-dihydroxybenzyl)ether [9,109]
**6.4**	32.38 ± 8.01 (a), 8.013 ± 3.06 (b), pM, *K*_i_	(E)-4-(3-bromo-4,5-dihydroxyphenyl)but-3-en-2-one [110]
**6.5**	24.38 ± 2.73 (a), 13.28 ± 0.07 (b), pM, *K*_i_	(E)-4-(2-bromo-4,5-dihydroxyphenyl)but-3-en-2-one [110]
**6.6**	19.02 ± 6.15 (a), 11.84 ± 3.47 (b), pM, *K*_i_	(E)-4-(2,3-dibromo-4,5-dihydroxyphenyl)but-3-en-2-one [110]
**6.7**	45.72 ± 3.30 (a), nM, *K*_i_	(4-bromo-2,5-dimethoxyphenyl) (phenyl)methanone [81]
**6.8**	37.29 ± 0.25 (a), nM, *K*_i_	(3-bromo-4-methoxyphenyl) (3,4-dimethoxyphenyl)methanone [81]
**6.9**	14.23 ± 1.99 (a), nM, *K*_i_	(3-bromo-4-methoxyphenyl) (2,3-dibromo-4-hydroxy-5-methoxyphenyl)methanone [81]
**6.10**	21.96 ± 7.60 (a), nM, *K*_i_	(2,3-dibromo-4-hydroxy-5-methoxyphenyl) (2,5-dibromo-4-methoxyphenyl)methanone [81]
**6.11**	8.94 ± 0.73 (a), nM, *K*_i_	(3-bromo-4-methoxyphenyl) (2,5-dibromo-4-methoxyphenyl)methanone [81]
**6.12**	59.45 ± 14.97 (a), nM, *K*_i_	(2,5-dibromo-4-methoxyphenyl) (phenyl)methanone [81]
**6.13**	27.55 ± 9.73 (a), nM, *K*_i_	(3-bromo-4-hydroxyphenyl) (3,4-dihydroxyphenyl)methanone [81]
**6.14**	1.53 ± 0.23 (a), 0.93 ± 0.20 (b), nM, *K*_i_	3,4-dibromo-5-((methylsulfonyl)methyl)benzene-1,2-diol [111]
**6.15**	0.84 ± 0.12 (a), 3.73 ± 1.03 (b), nM, *K*_i_	3,4,6-tribromo-5-((methylsulfonyl)methyl)benzene-1,2-diol [111]
**6.16**	10.82 (a), 22.35 (b), nM	(2-bromo-4-hydroxyphenyl) (phenyl)methanone [96]
**6.17**	13.07 (a), 30.13 (b), nM	(2-bromo-4-hydroxyphenyl)(4-hydroxyphenyl)methanone [96]
**6.18**	159.6 ± 21.9 (a), nM, *K*_i_	2,4-dibromo-4-(2,3-dibromo-4,5-dimethoxyphenyl)-3-methylbutanoic acid [46]

Notes: a, AChE; b, BChE; c, BACE1; d, GSK-3*β*; e, hMAO-A; unit for IC_50_ is μM and nM, unless labeled for *K*_i_ is nM and pM. f, D_4_R, and unit for EC_50_ is μM.

**Table 6:** Carbonic anhydrase inhibitory activity and names of compounds in Scheme 8.

No.	*K*_i_/IC_50_ (µM)	Names
**7.2.1**	86.4 (a), IC_50_	3,4,6-tribromo-5-(2,5-dibromo-3,4-dihydroxybenzyl)benzene-1,2-diol [115]
**7.2.2**	38.29 (a), IC_50_	5,5′-methylene bis(3,4,6-tribromo-benzene-1,2-diol) [115]
**7.2.3**	0.7 (a), IC_50_	(2-bromo-3,4-dihydroxyphenyl) (2,3-dibromo-4,5-dihydroxyphenyl)methanone [115]
**7.2.4**	1.13 (a), 1.84 (b), 12.24 (c), 3.41 (d)	2-bromo-4,6-bis(2,3-dibromo-4,5-dihydroxybenzyl)benzene-1,3,5-triol [116]
**7.2.5**	78.49 (a), 57.61 (b), 93.42 (c), 45.36 (d)	4-bromo-2,6-bis(2,3-dibromo-4,5-dimethoxybenzyl)-3,5-dimethoxyphenol [116]
**7.2.6**	0.56 (a), 1.08 (b), 1.67 (c), 0.59 (d)	2(R)-2-(2,3,6-tribromo-4,5-dihydroxybenzyl)cyclohexanone [41]
**7.2.7**	0.38 (a), 0.85 (b), 1.04 (c), 0.48 (d)	1R(S),2R(S)-2-(3-bromo-4,5 dimethoxybenzyl)cyclohexanol [41]
**7.2.8**	0.38 (a), 0.87 (b), 1.03 (c), 0.47 (d)	1(R)S,2S(R)-2-(3-bromo-4,5-dimethoxybenzyl)cyclohexanol [41]
**7.2.9**	0.41 (a), 0.93 (b), 1.12 (c), 0.51 (d)	1R(S),2R(S)-2-(2,3-dibromo-4,5-dimethoxybenzyl)cyclohexanol [41]
**7.2.10**	0.39 (a), 0.88 (b), 1.10 (c), 0.47 (d)	1(R)S,2S(R)-2-(2,3-dibromo-4,5-dimethoxybenzyl)cyclohexanol [41]
**7.2.11**	1.26 (a), 32.7 (c)	3,4-dibromo-5-(2,3-dibromo-4,5-dihydroxybenzyl)-6-(ethoxymethyl)benzene-1,2-diol [39]
**7.2.12**	0.65 (a), 13.7 (c)	(4,5-dihydroxy-2-methylphenyl) (3,4-dihydroxyphenyl)methanone [39]
**7.2.13**	0.74 (a), 18.5 (c)	(3-bromo-4,5-dihydroxy-2-methylphenyl) (3,4-dihydroxyphenyl)methanone [39]
**7.2.14**	0.83 (a), 22.6 (c)	(3-bromo-4,5-dihydroxy-2-methylphenyl) (2-bromo-4,5-dihydroxyphenyl)methanone [39]
**7.2.15**	0.92 (a), 28.5 (c)	(2-bromo-4,5-dihydroxyphenyl) (4,5-dihydroxy-2- methylphenyl)methanone [39]
**7.2.16**	9.24 (a), 7.87 (c),	(4-bromo-2, 5-dihydroxyphenyl) (3, 4-dihydroxyphenyl)methanone [96]
**7.2.17**	5.97 (a), 8.15 (c),	(4-bromo-2, 5-dihydroxyphenyl) (3, 4, 5-trihydroxyphenyl)methanone [96]
**7.2.18**	6.93 (a), 8.45 (c),	(2-bromo-4-hydroxyphenyl) (phenyl)methanone [96]
**7.2.19**	6.18 (a), 6.79 (c),	(2-bromo-4-hydroxyphenyl) (4-hydroxyphenyl)methanone [96]
**7.2.20**	5.58 (a), 7.61 (c),	(2-bromo-4-methoxyphenyl) (phenyl)methanone [96]
**7.2.21**	6.07 (a), 7.51 (c),	(2-bromo-4-methoxyphenyl) (4-methoxyphenyl)methanone [96]
**7.2.22**	8.4 ± 2.3 (c), 48.3 ± 1.3 (a), nM	(1R*,2R*,3R*)-ethyl 2-(2-bromo-4,5-dimethoxyphenyl)-3-methylcyclopropane-1-carboxylate [46]
**7.2.23**	10.7 ± 2.9 (c), nM	(1S*,2R*,3R*)-ethyl 2-(2,6-dibromo-3,4-dimethoxyphenyl)-3-methylcyclopropanecarboxylate [46]
**7.2.24**	7.8 ± 0.9 (c), nM	(1S*,2R*,3R*)-ethyl 2-methyl-3-(2,3,6-tribromo-4,5-dimethoxyphenyl)cyclopropanecarboxylate [46]
**7.2.25**	43.1 ± 1.7 (a), nM	(1R*,2R*,3R*)-2-(2-Bromo-4,5-dimethoxyphenyl)-3-methylcyclopropane-1-carboxylic acid [46]

Notes: a, hCA II; b, hCA IV; c, hCA I; d, hCA VI. Values are for *K*_i_, unless labeled as IC_50_.

**Table 7:** IC_50_ of G6PD inhibitory activity and names of compounds in Scheme 9.

No.	IC_50_ (μM)	Names
**7.3.1**	76.6 ± 0.1 (a)	3,5-dibromo-4-hydroxybenzaldehyde [117]
**7.3.2**	4.01 ± 0.3 (a),	3,4-dibromo-5-(2-bromo-3,4-dihydroxy-6-(hydroxymethyl)benzyl)benzene-1,2-diol [117]
**7.3.3**	0.85 ± 0.1 (a)	Bis(2,3-dibromo-4,5-dihydroxybenzyl)ether [117]
**7.3.4**	321 ± 18 (a)	3,4-dibromo-5-(butoxymethyl)benzene-1,2-diol [118]
**7.3.5**	0.97 ± 0.1 (a)	Bis-(2,3,6-tribromo-4,5-dihydroxybenzyl methyl ether) [118]
**7.3.6**	0.85 ± 0.1 (a)	5,5’-(oxybis(methylene))bis(3,4-dibromobenzene-1,2-diol) [118]
**7.3.7**	0.47 ± 0.03 (a)	5,5’-methylenebis(3,4-dibromobenzene-1,2-diol) [118]
**7.3.8**	0.53 ± 0.18 (a)	3,4-dibromo-5-(2-bromo-3,4-dihydroxy-6-(methoxymethyl)benzyl)benzene-1,2-diol [118]

Notes: a, *Leuconostoc mesenteroides* G6PD; b, *Saccharomyces cerevisiae* G6PD.

## Abbreviations

A549: human lung adenocarcinoma epithelial cell line; ABTS, 2,2′-azinobis (3-ethylbenzothiazoline-6-sulfonic acid) diammonium salt; AChE, acetylcholinesterase; AD, Alzheimer’s disease; ADME, absorption, distribution, metabolism, and excretion; APN, acute pyelonephritis; AR, aldose reductase; BACE1, *β*-site amyloid precursor protein cleaving enzyme 1; BChE, butyrylcholinesterase; Bel7402, human hepatoma cell line; B16F10, malignant melanoma cell line; BHT, butylated hydroxytoluene; BPs, bromophenols; CA, carbonic anhydrase; Caco2, human clonal colon adenocarcinoma cell line; C2C12, mouse myoblast cell line; ChEs, cholinesterases; CPT-1B, carnitine palmitoyl transferase 1B; 95D, human highly metastatic lung cancer cell line; D_3_/D_4_R, dopamine D_3_/D_4_ receptor; DPPH, 1,1-diphenyl-2-picryl hydrazyl; ERK, extracellular signal-regulated kinase; FABP3, fatty acid binding protein 3; FAK, focal adhesion kinase; G6PD, glucose 6-phosphate dehydrogenase; GSH, glutathione; GSK-3*β*, glycogen synthase kinase-3*β*; HbA1c, hemoglobin A1c; HCC-1937, human breast cancer cell line; HCT-8, human colorectal carcinoma cell line; HCT-116, human colorectal carcinoma cell line; HeLa, human cervical carcinoma cell line; HepG2, human hepatocellular carcinoma cell line; HgA, hemagglutinin A; hMAO-A, human monoamine oxidase-A; HUVEC, human umbilical vein endothelial cell line; IgE, immunoglobulin E; IHNV, infectious hematopoietic necrosis virus; IKK, iκB kinase; IL-6, interleukin-6; IL-1β, interleukin-1β; IPNV, infectious pancreatic necrosis virus; K562, human myelogenous leukemia cell line; Keap1, kelch-like ECH-associated protein 1; MAPK, mitogen-activated protein kinase; MCF-7, human breast adenocarcinoma cell line; MDB-MB-231, human breast cancer cell line; MDR-TB, multidrug resistant-tuberculosis; MIC, minimum inhibitory concentration; mTOR, mammalian target of rapamycin; NCI-H460, human large cell lung cancer cell line; NF-κB, nuclear factor kappa-light-chain-enhancer of activated B cells; Nrf2, nuclear factor erythroid 2-related factor 2; PBDEs, polybrominated diphenyl ethers; PD, Parkinson’s disease; PGE2, prostaglandin E2; PON1, paraoxonase-1; PI3K, phosphoinositide 3 kinase; PTPs, protein tyrosine phosphatases; PTP1B, tyrosine phosphatase 1B; RAW 264.7, leukemia cells in mouse macrophage; RBPs, RNA-binding proteins; Rgp, gingipain R; RKO, human colon adenocarcinoma cell line; ROS, reactive oxygen species; SAR, structure activity relationships; SK-OV-3, human ovarian cancer cell line; SMMC-7721, human hepatocellular carcinoma cell line; STAT1, signal transducer and activator of transcription 1; TEAC, trolox equivalent antioxidant capacity; TNF-α, tumor necrosis factor-α; U87, human glioma cell line; UGTs, UDP-glucuronosyltransferases; UVB, ultraviolet B; VEGF, vascular endothelial growth factor; Vero, African green monkeys kidney cells.
